# Ocular sarcoidosis in adults and children: update on clinical manifestation and diagnosis

**DOI:** 10.1186/s12348-023-00364-z

**Published:** 2023-09-18

**Authors:** Magdalena Bazewicz, Jarmila Heissigerova, Carlos Pavesio, François Willermain, Janusz Skrzypecki

**Affiliations:** 1 Department of Ophthalmology, Centre Hospitalier Universitaire Saint-Pierre, Brussels, Belgium; 2 Department of Ophthalmology, Centre Hospitalier Universitaire Brugmann, Brussels, Belgium; 3 Department of Ophthalmology, First Faculty of Medicine, Charles University and General University Hospital in Prague, Prague, Czech Republic; 4 Uveitis Service, Moorfields Eye Hospital, National Health Service Foundation Trust London, London, UK; 5 University College London, London, UK; 6 Université Libre de Bruxelles (ULB), Brussels, Belgium; 7 Department of Ophthalmology, Independent Public University Eye Hospital, Warsaw, Poland; 8 Department of Experimental Physiology and Pathophysiology, Medical University of Warsaw, Warsaw, Poland

**Keywords:** Uveitis, Sarcoidosis, Diagnosis, Criteria, Biomarkers, Children

## Abstract

Sarcoidosis-associated uveitis, is the predominant ocular sarcoidosis presentation, which affects both adults and children. For adults, international ocular sarcoidosis criteria (IWOS) and sarcoidosis-associated uveitis criteria (SUN) are defined. However, for children they are not yet established internationally. Due to the specificity of pediatric manifestations of sarcoidosis, this task is even more challenging. In children, sarcoidosis is subdivided into Blau syndrome and early-onset sarcoidosis (BS/EOS) affecting younger children (< 5 years) and the one affecting older children with clinical presentation resembling adults. Differential diagnosis, clinical work-up as well as diagnostic criteria should be adapted to each age group. In this article, we review the clinical manifestation of sarcoidosis-associated uveitis in adults and children and the sensitivity and specificity of various ocular sarcoidosis diagnostic modalities, including chest X-ray and CT, FDG PET-CT, gallium-67 scintigraphy, bronchoalveolar lavage fluid, genetic testing for NOD2 mutations and serum biomarkers, such as ACE, lysozyme and IL2R.

## Introduction

Sarcoidosis is a rare inflammatory condition of unknown cause that affects both adults and children. Its prevalence in adults ranges from 8.1/100 000 in Caucasians to 17.8/100 000 in African Americans. Notably, the disease is less common in children with a prevalence of 0.22–0.27/100000 (Danish study) [1].

The hallmark of the disease is the presence of non-caseating granulomas in affected tissues. Nevertheless, systemic presentation varies among age groups, which often leads to delayed diagnosis in younger patients. It is of note that sarcoidosis in children < 5 years old does not typically involve lungs but skin, joints, and eyes [2, 3].

Ophthalmic manifestations of sarcoidosis can involve any part of the eye and its adnexa in the inflammatory process [4]. The prevalence of ocular involvement in patients diagnosed with systemic sarcoidosis ranges from 13 to 79% [4]. Furthermore, ocular involvement remains the presenting symptom in 30–40% of patients diagnosed with systemic sarcoidosis [5, 6]. The most common ocular manifestation of sarcoidosis is uveitis and is reported in up to 70% of cases [4]. International Workshop on Ocular Sarcoidosis (IWOS) clinical criteria for ocular sarcoidosis are referring only to uveitis and not to other ophthalmic manifestations. Following nomenclature used by IWOS and many other authors, in this review ‘ocular sarcoidosis’ refers only to sarcoidosis-associated uveitis.

Considering the above, certain patients diagnosed with uveitis e.g. patients with granulomatous or posterior uveitis should undergo sarcoidosis screening as a routine workup. However, a definite diagnosis is often difficult to obtain as according to the revised IWOS Criteria for diagnosis of ocular sarcoidosis a biopsy of the lesion is required [7]. It is feasible for the skin and lymph node involvement but is often avoided in the case of vital organs i.e. lungs, heart, or liver [7]. Therefore, a high-level of clinical suspicion and a thorough slit-lamp examination are essential for setting a presumed or probable diagnosis [7].

This clinical scenario is even more complex in children. The lack of a typical pattern of systemic presentation and the absence of standardized guidelines on ocular sarcoidosis in children makes a clinical diagnosis of ocular sarcoidosis difficult [3].

Here, we present overlapping as well as distinguishing features of ocular sarcoidosis in children and adults followed by a differential diagnosis and novel diagnostic approach. The contrast between features of the disease across various age groups aims to increase clinicians' awareness regarding this diagnosis and ensure a timely diagnosis for more patients, particularly those under 5 years of age.

## Methods

We utilized the Pubmed database to search for relevant publications. Manuscripts published between 2013 and 2022 were utilized as the primary source of data. Ocular sarcoidosis, sarcoidosis, early onset sarcoidosis, Blau syndrome, and uveitis served as primary search terms.

## Systemic sarcoidosis

### Adults

There are no firmly established guidelines dedicated to the diagnosis of sarcoidosis. However, it is agreed that the disease is deemed highly probable in an individual with typical clinical features, non-caseating granulomas in the histopathological examination, and in whom other granulomatous diseases have been ruled out [8].

The 2014 guidelines issued by the World Association of Sarcoidosis and other Granulomatous Diseases point out that certain clinical manifestations and imaging or laboratory findings are suggestive of the disease and these patients should undergo sarcoidosis screening [9]. Clinical manifestations include: Lofgren syndrome (bilateral hilar adenopathy, erythema nodosum, and/or arthritis), Heerfordt-Waldenström syndrome (rare subacute variant of sarcoidosis, characterized by enlargement of the parotid or salivary glands, facial nerve paralysis and anterior uveitis) [10], lupus pernio, uveitis, optic neuritis, erythema nodosum. Nonspecific benign lymphoepithelial lesion or Mikulicz's disease or syndrome is a type of benign enlargement of the parotid and/or lacrimal glands, which has been described also in sarcoidosis. Virtually any organ and system can be involved in the process including the central nervous system, liver, kidneys, spleen, or muscles [9]. Imaging features include: bilateral hilar adenopathy, perilymphatic nodules, gadolinium enhancement on MRI, osteolysis, trabecular bone pattern, bone cysts and contrast uptake by the parotid; laboratory findings include hypercalcemia or hypercalciuria [9]. It is of note that the conditions listed above do not exhaust all possible sites involved in sarcoidosis.

Considering that the clinical picture of the disease is non-specific, a biopsy of the lesion is usually endorsed by sarcoidosis experts [11]. However, as sarcoid granulomas do not have any specific features that would allow them to distinguish them from other non-necrotizing granulomas, the exclusion of other diseases with similar histopathology is usually required to confirm the diagnosis of sarcoidosis [12]. The differential diagnoses of systemic sarcoidosis should include infectious and non-infectious causes [13]. The detailed differential of systemic sarcoidosis remains beyond the scope of this review, however, the most common mimickers include tuberculosis and other mycobacteria, fungal infections, and a range of non-infectious diseases including vasculitis or lymphoma [14]. For a more thorough list of differential diagnoses of systemic sarcoidosis, the reader is referred to dedicated sources [13–17].

### Children > 5 years

The course of systemic sarcoidosis in children older than 5 years is similar to the adult disease described above [3, 18]. Clinical symptoms include fever of unknown origin and malaise accompanied by hilar adenopathy and lung changes [18]. Interestingly, peripheral lymph nodes involvement is more common in children (40–70%) than in adults (4.8%) [1, 2].

Systemic sarcoidosis usually affects teenagers with a mean age of 13–15 years. Notably, its prevalence is much lower than in adults and a recent Danish study showed that it equals 0.22–0.27/100 000 [1]. Sarcoidosis in children older than 5 years has usually a less severe course than in adults. It is estimated that approximately 25% of adults and 12% of children develop chronic or progressive disease [19, 20].

### Children < 5 years

Sarcoidosis in children younger than 5 years is usually considered separately and is labeled as Blau Syndrome when there is a family history proving an autosomal dominant trait or Early Onset Sarcoidosis (EOS) when no family history is evident [21]. Children under 5 typically present with a triad of a rash, polyarthritis, and uveitis [22]. Although any organ can be involved in the inflammatory process, lungs are typically spared [23]. BS and EOS are respectively defined as the familial and sporadic forms of the same pediatric noncaseating granulomatous autoinflammatory disease [24] and are shown to share a common genetic etiology [25]. Although, traditionally, Blau syndrome is recognized if there is a family history of the disease, and EOS if the mutation is sporadic [21], this traditional naming is not always followed, as authors of some recent publications [26–29] use the name Blau Syndrome for both familial and sporadic mutations.

Notably, Blau syndrome and EOS are a result of Nucleotide-binding oligomerization domain-containing protein 2 (NOD2), previously known as CARD15 gene mutation [30]. NOD2 serves as an intracellular pattern recognition receptor that activates the immune system in response to muramyl dipeptide, a constituent of the cell wall of certain bacteria. Therefore, the gain mutation of this gene leads to the overactivation of the immune system [30].

Data from the Blau syndrome registry shows that patients with Blau syndrome develop rash, arthritis, and uveitis with a median age of 1.1 years, 2 years, and 4.4 years respectively [31]. Furthermore, 30 out of 31 patients included in a published report required systemic medication to control the disease [26, 31]**.** Other expanded manifestations of BS/EOS are fever, pneumonitis, bronchial granulomas, hepatosplenomegaly, hepatic granulomas, sialadenitis, erythema nodosum, leukocytoclastic vasculitis, transient neuropathies, arterial hypertension, pericarditis, pulmonary embolism, granulomatous glomerular and interstitial nephritis, and chronic renal failure [21]. Expanded manifestations beyond the classical triad were observed in 52% patients [31]. Neurologic involvement is infrequent in BS/EOS. Typical central nervous system manifestations seen in adult sarcoidosis, namely meningeal and white matter disease have not been described in Blau until 2021 [21, 32].

## Ophthalmic manifestations of sarcoidosis and ocular sarcoidosis

Ophthalmic manifestations of sarcoidosis affect virtually any part of the eye and its adnexa. The most common form includes uveitis (anterior, intermediate, posterior) and conjunctival granuloma. The former may lead to significant visual disability, whereas the latter is usually asymptomatic. Other ophthalmic manifestations of sarcoidosis consist of dacryoadenitis, orbital inflammation, eyelid granuloma, madarosis, poliosis, keratitis, and optic neuritis [33].

Coulon et al. reported that among 194 adult patients with biopsy-proven and presumed uveitis only 9% had additionally other ocular involvement, including conjunctival node (2%), scleritis (1%), episcleritis (1%), optic neuropathy (5%), dacryoadenitis (0,5%) [34].

Ocular sarcoidosis might thus globally refer to any inflammation of the eye and its adnexa [4] and can be divided in ocular surface, intraocular (uveitis), adnexal, orbital and neuro-ophthalmological manifestation. However, as mentioned above, in this review, ‘ocular sarcoidosis’ follows nomenclature used in the cited studies and practically refers to sarcoidosis-associated uveitis.

Ocular sarcoidosis as well as systemic sarcoidosis show some differences among the ethnic groups. Systemic involvement of sarcoidosis is more frequent in North Africans than in White Europeans, who show a higher frequency of isolated ocular involvement at onset and during follow-up [34]. Anterior uveitis is more frequent in Afro-Caribbeans (59.1%) [34]. Caucasian sarcoid uveitis patients are older at presentation (48 vs 41 years; *P* = 0.009) and have less granulomatous anterior uveitis (26.4% vs 51.7%; *P* < 0.001) [35]. Afro-Caribbeans and North Africans have first ocular manifestation of the disease earlier than White Europeans (*p* < 0.001), respectively 34.3, 43.1 and 57.8 years [35].

## Uveitis

### Adults

Features of sarcoidosis-associated uveitis are included in numerous cohort studies [7, 36, 37] as well as in the international diagnostic criteria such as revised diagnostic criteria of ocular sarcoidosis by IWOS (Table 1) [7] and criteria for sarcoidosis-associated uveitis by Standardization of Uveitis Nomenclature (SUN) Working Group [38] (Table 2). Characteristics and common features of sarcoid uveitis in adults are presented in Table 3 which include the biggest recent ophthalmologic studies in adult patients with sarcoidosis.

**Table 1:** Revised International Workshop on ocular sarcoidosis (IWOS) criteria for the diagnosis of ocular sarcoidosis (OS)^7^, published in 2019

I. Other causes of granulomatous uveitis must be ruled out
II. Intraocular clinical signs suggestive of OS
1. Mutton-fat keratic precipitates (large and small) and/or iris nodules at pupillary margin (Koeppe) or in stroma (Busacca)
2. Trabecular meshwork nodules and/or tent-shaped peripheral anterior synechia
3. Snowballs/string of pearls vitreous opacities
4. Multiple chorioretinal peripheral lesions (active and atrophic)
5. Nodular and/or segmental periphlebitis (± candle wax drippings) and/or macroaneurysm in an inflamed eye
6. Optic disc nodule(s)/granuloma(s) and/or solitary choroidal nodule
7. Bilaterality (assessed by ophthalmological examination including ocular imaging showing subclinical inflammation)
III. Systemic investigation results in suspected OS
1. Bilateral hilar lymphadenopathy (BHL) by chest X-ray and/or chest computed CT scan
2. Negative tuberculin test or interferon-gamma releasing assays
3. Elevated serum ACE
4. Elevated serum lysozyme
5. Elevated CD4/CD8 ratio (> 3.5) in bronchoalveolar lavage fluid
6. Abnormal accumulation of gallium-67 scintigraphy or 18F-fluorodeoxyglucose positron emission tomography imaging
7. Lymphopenia
8. Parenchymal lung changes consistent with sarcoidosis, as determined by pulmonologists or radiologists
IV. Diagnostic criteria
Definite OS: diagnosis supported by biopsy with compatible uveitis
Presumed OS: diagnosis not supported by biopsy, but BHL present with two intraocular signs Probable OS: diagnosis not supported by biopsy and BHL absent,but three intraocular signs and two systemic investigations selected from two to eight are present

Reproduced from Br J Ophthalmol., Mochizuki M, et al., 103(10):1418–22, copyright 2019 with permission from BMJ Publishing Group Ltd

**Table 2:** The criteria for sarcoidosis-associated uveitis by Standardization of Uveitis Nomenclature (SUN) Working Group^33^, published in 2021

1. Compatible uveitic picture, either
a. Anterior uveitis OR
b. Intermediate or anterior/intermediate uveitis OR
c. Posterior uveitis with either choroiditis (paucifocal choroidal nodule(s) or multifocal choroiditis) OR
d. Panuveitis with choroiditis or retinal vascular sheathing or retinal vascular occlusion
AND
2. Evidence of sarcoidosis, either
a. Tissue biopsy demonstrating non-caseating granulomata OR
b. Bilateral hilar adenopathy on chest imaging
Exclusions
1. Positive serology for syphilis using a treponemal test
2. Evidence of infection with Mycobacterium tuberculosis^a^ either
a. Histologically- or microbiologically-confirmed infection with M. tuberculosis^b^OR
b. Positive interferon-Ɣ release assay (IGRA)^c^ OR
c. Positive tuberculin skin test^d^

Reprinted from Am J Ophthalmol. 228, Standardization of Uveitis Nomenclature Working G. Classification Criteria for Sarcoidosis-Associated Uveitis. p: 220–30., Copyright 2021, with permission from Elsevier

^a^Routine exclusion of tuberculosis is not required in areas where tuberculosis is non-endemic but should be performed in areas where tuberculosis is endemic or in tuberculosis-exposed patients. With evidence of latent tuberculosis in a patient with a uveitic syndrome compatible with either sarcoidosis or tubercular uveitis and bilateral hilar adenopathy, the classification as sarcoid uveitis can be made only with biopsy confirmation of sarcoidosis (and therefore exclusion of tuberculosis)

^b^E.g. biopsy, fluorochrome stain, culture, or polymerase chain reaction based assay

^c^E.g. Quantiferon-gold or T-spot

^d^E.g. Purified protein derivative (PPD) skin test; a positive result should be > 10 mm induration

**Table 3:** Characteristics and common features of sarcoid uveitis in adults, children and Blau Syndrome/ Early Onset Sarcoidosis (BS/EOS)

	Study	nbr of patients in the study with ocular manifestation of sarcoidosis or BS/EOS	Number of all patients in the study	Age, years, median of patients’ age with ocular manifestations of sarcoidosis or BS/EOS; for BS/EOS median age at ocular onset and age at study baseline	Bilateral	Granulomatous	Mutton-fat keratic precipitates	Mutton-fat KPs, iris nodules, or both	Non granulomatous
Adults	SUN, 2021	278^b^	2684	49 (IQR 39–61)	82%	-	23%	35%	-
	Acharya,2018	167^c^	884	49 (IQR 39—60)	86%	-	35%	46%	-
	Coulon, 2019	194^d^	194	52.1 ± 17.8	77.8%	60%	-	-	-
	Niederer, 2021	362^e^	362	46 (IQR 35–57)	87%	48%	-	-	-
	Allegri, 2022	235	235	mean age 52	85%	52%	-	-	-
Children > 5 years	Choi, 2011	13^f^	460	12 (range: 5 to 16)	-	31%	-	-	62%
	Morelle, 2019	9^b^	147	-, mean age 10.5 (range: 7–14)	100%	23%	-	-	-
	Waduthantri S, 2021	8^ g^	73	mean age 12 (range: 5–15)	-	-	-	-	-
Blau Syndrome / Early Onset Sarcoidosis	Sarens, 2018	38	50	5 (range 0,5- 48); at study baseline: 17 (range 2–56)	97%	-	-	-	-
	Kumrah, 2022	9	11	4 (range: 2–26); median age at dignosis: 9 (range: 2–26)	100%	44%	-	11% only iris nodules specified	11%
	Matsuda, 2020	38	50	-, -; all patients in the study: mean 26.7 (range 0–61)	-	-	-	-	-
	Wu,2019	7	7	5,5 (range 2-24y), at study baseline: 10,5 (range: 4–34)	-	-	-	-	-
	Babu, 2020	7	7	-, at study baseline: 9 (range 2,5–25)	71%^i^	71%^j^	14%	-	-
	Rosé, 2015	25	31	4.4 (range 0,5–22); at study baseline 16.5 (range: 1.9–58)	96%	-	-	-	-

	No keratic precipitates	Anterior^a^ uveitis	Intermediate^a^ uveitis	Posterior^a^ uveitis	Panuveitis ^a^	Multifocal choroiditis	Retinal vascular inflammation^h^	Solitary choroidal nodule (optic disc nodules or granulomas)
Adults	52%	40%	19%	4%	37%	30%	18%	2% (-)
	-	20%	3%	9%	67%	45%	36%	3% (4%)
	-	34%	10%	7%	49%	40%	30%	-
	-	-	-	-	-	43%	21%	11% isolated choroidal or optic nerve granuloma
	-	52%	10%	29%	8%	-	-	-
Children > 5 years	8%	-	-	-	-	54%	31%	-
	-	77%	-	-	23%	-	-	-
	-	12.50%	-	25%	62.50%	-	-	-
Blau Syndrome / Early Onset Sarcoidosis	-	29%	-	-	51%	39%	0%	- (12%) peripapillary nodules
	-	33%	-	-	67%	67%	11%	-
	-	-	-	-	-	-	-	-
	-	14%	-	-	85%	-	-	-
	-	29%	-	-	43%	-	-	14% (-)
	-	-	-	-	-	-	-	-

*IQR* interquartile range; data not available

^a^anatomic class (SUN)/ type by anatomic location (Acharya, 2018)

^b^sarcoidosis-associated uveitis

^c^including 98 patients with definite ocular sarcoidosis and 69 patients with presumed ocular sarcoidosis according to IWOS 2009 criteria

^d^including 145 with biopsy-proven and 49 with presumed sarcoid uveitis following the WASOG/ATS/ERS criteria

^e^including definite or presumed ocular sarcoidosis according to IWOS 2009 criteria

^f^including 4 definite, 3 presumed, or 6 probable sarcoidosis according to selfestblished scoring system

^g^8 patients with presumed ocular sarcoidosis and 2 patients with sarcoid scleritis

^h^refered to as: retinal vascular sheathing or periphebitis or vasculitis

^i^at least 71%, no data available on bilaterality for 2 other patients with only conjunctival granulomas

^j^and additionally 29% with only conjunctival granulomas

- no data available

Uveitis in adults presents typically as a bilateral and chronic disease. It is predominantly either panuveitis (8–67%) or anterior uveitis (20–52%) and it varies among the studies. Intermediate uveitis is less frequent (3–19%) and isolated posterior uveitis is rare (4–29%) [34, 38–40].

**Fig. 1:**
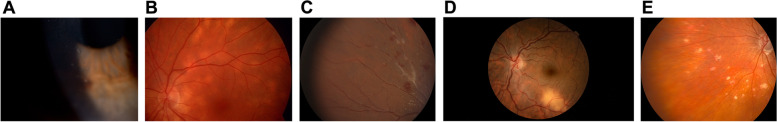
Anterior and posterior uveitis findings in adult patients with definite or presumed ocular sarcoidosis. **A** Color photography of granulomatous keratic precipitates type mutton-fat in patient with definite ocular sarcoidosis proven by biopsy from lacrimal gland. **B** Color fundus photography of multifocal choroiditis in the left eye of a patient with definite ocular sarcoidosis proven by biopsy of cervical lymph nodes. **C** Fundus color photography showing periphlebitis with perivenous sheating and retinal hemorrages in a patient with presumed sarcoidosis. **D** Color fundus photography of choroidal granuloma in the left eye of a patient with definite ocular sarcoidosis proven by biopsy of intrathoracic lymph nodes and lung. **E** Fundus color photography showing peripheral chorioretinal lesions in a patient with presumed sarcoidosis

Anterior segment inflammation is granulomatous in 48–60% [34, 35, 40]. The presence of mutton-fat keratic precipitates (Fig. 1A) and/or iris stromal nodules is observed in up to 46% of patients [39, 40]. 27% of patients with sarcoid uveitis present also with posterior synechiae [38]. Additional features of anterior sarcoid uveitis include trabecular meshwork nodules and tent-like anterior synechiae, which can be observed in 18% and 35% of patients respectively [39].

Although intermediate uveitis is not frequent, vitreous involvement is often observed with snowballs or string of pearls in 17–50% of patients [38, 39].

**Fig. 2:**
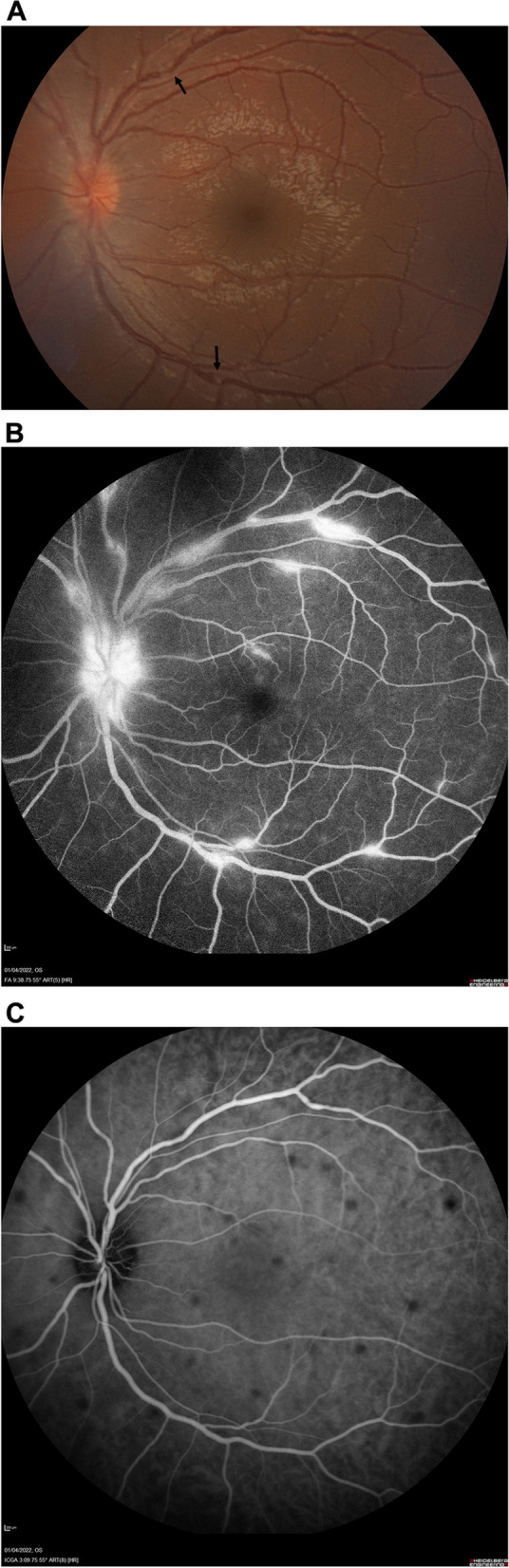
Multimodal imaging of posterior uveitis in a patient with presumed ocular sarcoidosis. The vasculitis might normally barely be seen in that particular case and is strongly highlighted by the fluorescein angiogram. **A** Color fundus photography of the left eye showing hyperemic optic disc and barely visible vasculitis. Black arrows: barely visible vasculitis. **B** Fundus fluorescein angiography of the left eye showing hot disc and active vasculitis (**C**) Indocyanine green angiography of the left eye showing hypocyanescent spots

The most common manifestation of sarcoid-associated uveitis in the posterior segment is multifocal choroiditis (Fig. 1B) followed by retinal vascular sheathing/periphlebitis (Figs. 1C and 2A, B) and rarely choroidal or optic disc granuloma (Fig. 1D) (Table 3).

### Children > 5 years

The available data concerning children is very limited. In Table 3 most recent and largest studies were included. Among the published data, in the last 12 years only three studies present each more than 5 pediatric patients with detailed description of ocular involvement in sarcoidosis.

The paucity of data allows only for preliminary conclusions. Granulomatous anterior uveitis is present in 23–31% of pediatric patients above 5 years old. One study indicates non-granulomatous anterior uveitis as more prevalent in children than granulomatous anterior uveitis [41]. Inflammation in the anterior segment is the most frequent manifestation (77%), followed by panuveitis [42] in the European population (France). However, in a multi-ethnic Asian population (Singapore) sarcoidosis-associated panuveitis is the most frequent (63%), followed by posterior uveitis [43]. As in adults, multifocal choroiditis and periphlebitis are the most prevalent forms of posterior segment involvement [41].

### Children < 5 years

Multicenter studies on patients with Blau syndrome reported a 76–81% prevalence of ocular involvement (with bilateral disease in 96–97% of patients) [26, 31].

**Fig. 3:**
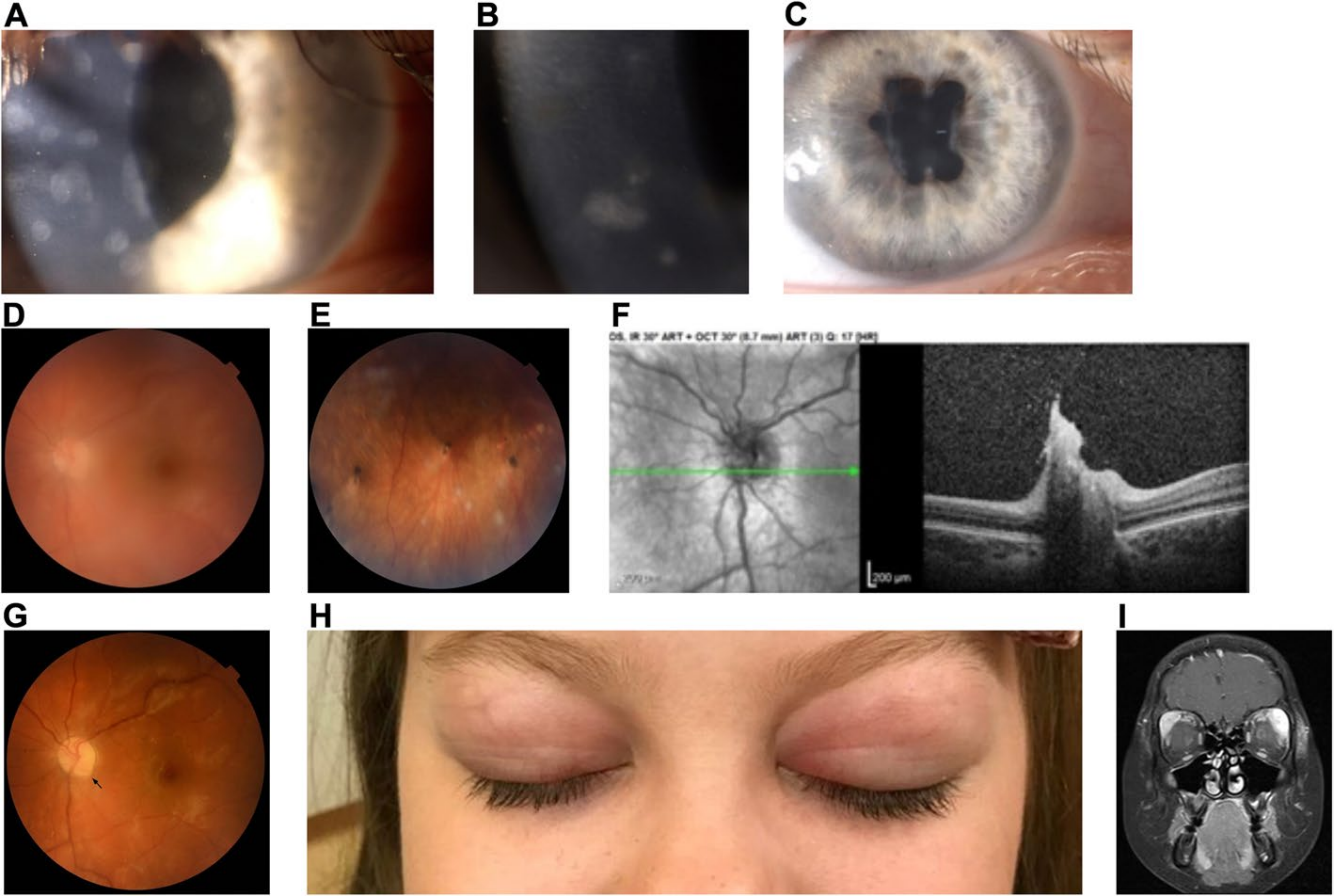
A pediatric female patient with suspected Blau Syndrome with de novo NOD2 mutation was followed for 10 years (2–12 y.o.). During the course of the disease, bilateral corneal old and new active subepithelial nummular infiltrates were noted and visualized here on color photographs of the right eye (**A**) and (**B**). The patient also developed bilateral posterior synechiae visualized here on color photograph of the right eye (**C**). Her fundus examination revealed bilateral vitritis and multifocal choroiditis with active and inactive peripheral lesions visualized on the left eye fundus color photography (**D**, **E**). Left eye optic disc granuloma, which appeared during relapse on adalimumab, was documented on the OCT (**F**) and later on the fundus color photography (**G**) after vitritis resolution following treatment with infliximab. Several years later, during another relapse initiated by switching to anakinra treatment, the patient developed Mikulicz’s syndrome with enlargement of bilateral lacrimal glands visualized on (**H**) color photography and on (**I**) brain MRI, coronal plane. The disease is now controlled with JAK-1 inhibitor baricitinib. Black arrow: optic disc granuloma

The typical ocular presentation in Blau syndrome is bilateral panuveitis observed in 43–85% of patients [26] (Table 3) (Fig. 3). Anterior uveitis occurred in 14–33% of patients. Intermediate and posterior uveitis, as predominant sites of inflammation, were not noticed. Some patients present with mutton-fat keratic precipitates [44] and some with nummular corneal infiltrates, white when new and active, almost transparent when inactive (Fig. 3A and B). Sarens et al. reported that chorioretinal disease, optic disc involvement and macular edema were observed at baseline in 39%, 29%, and 11% of patients respectively [26].

Available studies show that uveitis in Blau syndrome is resistant to anti-inflammatory treatment [26]. Despite treatment with local steroids and systemic immunosuppressive agents, Sarens et al. did not observe any statistically significant decrease in inflammation over a 3-year follow-up period [26]. Furthermore, in addition to posterior segment inflammation, complications related to anterior segment involvement led to significant ocular morbidity [26]. The authors of the study reported the prevalence of band keratopathy, posterior synechiae, and cataracts as 21%, 45% (Fig. 3C), and 55% respectively [26].

## Differential diagnosis

### Adults

Differential diagnosis depends largely on the uveitis presentation, especially the main site involved.

For the most typical presentation of sarcoidosis, bilateral granulomatous posterior/panuveitis (Table 3), the main differential diagnosis includes tuberculosis and syphilis.

Some posterior presentations of sarcoidosis can resemble Birdshot, APMPPE, recurrent VKH, sympathetic ophthalmia, intraocular lymphoma or tubulointerstitial nephritis and uveitis (TINU) syndrome [45].

Other causes of granulomatous uveitis may also be included especially in cases of atypical presentations or in immunocompromised patients: toxoplasmosis, lyme, cat scratch disease, cryptococcosis (in immunocompromised patients) or even endophthalmitis [46].

Rarely sarcoid uveitis can have less typical presentations eg. unilateral (16%) [39] or non-granulomatous, which will then open up the differential diagnosis to include other conditions such as viral uveitis in case of unilateral granulomatous anterior uveitis.

Non-caseating granuloma remains a histopathological hallmark for sarcoidosis and is included in diagnostic criteria of definite ocular sarcoidosis [7] and sarcoidosis-associated uveitis [38]. However, caseation in the granulomas in sarcoidosis can also occur, which may complicate differential diagnosis with tuberculosis [47, 48],

### Children > 5 years

The typical presentation of ocular sarcoidosis in this age group is bilateral anterior uveitis, which can be either granulomatous or non-granulomatous (Table 3.)

In case of anterior granulomatous as well as non-granulomatous uveitis, juvenile idiopathic arthritis (JIA) should be considered in the differential diagnosis. Indeed, although granulomatous uveitis in JIA really makes you wonder about the diagnosis, at least two papers mention that JIA-associated uveitis can be granulomatous [49, 50]. Furthermore, uveitis in sarcoidosis can also present as non-granulomatous anterior uveitis [41].

Also, TINU, although known mainly as anterior non-granulomatous uveitis, can present as granulomatous uveitis with subclinical choroidal involvement [45].

In case of bilateral granulomatous panuveitis/posterior uveitis, the main differential diagnosis is ocular tuberculosis.

In adolescents, as in adults, syphilis can be considered in differential diagnosis, although pure choroidal disease will not be syphilis.

### Children < 5y

In Blau syndrome, a typical association of uveitis with arthritis and skin rash can guide the diagnosis. It is worth noticing that skin involvement and arthritis in EOS differ from the ones in JIA (previously called JRA) [3]. However, an BS/EOS patient without joint involvement has also been reported [51]. In Blau syndrome, differential diagnosis with JIA, Behçet’s disease, ocular tuberculosis and ocular sarcoidosis was proposed [27, 28].

## Diagnostic modalities

Diagnosis of ocular sarcoidosis recommended by IWOS, unless biopsy-proven, relies on various clinical and investigational criteria (Table 1). Most of the studies supporting these criteria or other potential sarcoidosis biomarkers included only adults. In clinical practice, in the absence of guidelines dedicated for children, the ocular sarcoidosis adults’ criteria are used also for the pediatric population. However, literature on ocular sarcoidosis biomarkers in children is very scarce or missing. Here, we resume the available data on systemic imaging modalities and biomarkers for ocular sarcoidosis relating to adults as well as to children.

### Chest X-ray and chest computed tomography (CT) scan

#### Adults

Chest X-ray and/or chest computed CT scan are the mainstay imaging examinations in the diagnosis of granulomatous uveitis. Their role is to detect pulmonary changes characteristic for pulmonary sarcoidosis or tuberculosis. The findings that can support sarcoidosis diagnosis are bilateral hilar lymphadenopathy (BHL) and/or parenchymal lung changes. High resolution CT or CT with contrast were also described in patients with ocular sarcoidosis [52, 53].

In diagnosing presumed or probable ocular sarcoidosis, BHL and parenchymal lung changes are the criteria recognized by IWOS 2019. Chest X-ray and CT differ not only in their sensitivity and specificity in detecting this sarcoidosis related pulmonary features but also in availability and cost of the examination.

Chest X-ray still plays a role and its biggest advantage is low cost and availability. Chest X-ray was shown to be the second, after tuberculin skin test, most contributory investigation among the first step's systematic tests in patients with uveitis. Chest CT was placed among the second step's systematic tests in patients with uveitis, and among these it was a second most often contributory investigation, after HLA-B27 [54].

Chest CT was shown in many studies to have higher sensitivity (with similar specificity) in detecting BHL than chest X-ray. Nevertheless, for diagnosis of ocular sarcoidosis, even chest radiograph with its sensitivity 68% accompanied by specificity 96%, had high enough sensitivity to be considered as sufficient evidence of sarcoidosis among patients with uveitis [39]. In the same study, sensitivity of BHL on chest CT scan in patients with negative chest radiography results was 73% with high specificity 95%. Other recent studies showed lower chest X-ray BHL sensitivities in OS (57,1 -57,6%) (Burger 2021 57,1%, Niederer RL 2019, 57.6%) with 100% specificity. For comparison the chest CT scans BHL sensitivity for OS was higher and ranged from 85.7% [55] to 98.0% [35] with a specificity of 95.5%-100% [35, 55].

BHL, either by chest CT scans or chest X-ray, was found to be the most sensitive investigational finding in a study evaluating first IWOS ocular sarcoidosis criteria from 2009 [39].

Interestingly, BHL frequency is shown to significantly differ in an age-related manner in patients with uveitis associated with sarcoidosis. In Japan, older patients (> 65 years) with OS had BHL detectable in 52% and younger patients (≤ 65 years) in 78% of cases [56].

CT is also more sensitive in detecting parenchymal lung changes, another typical manifestation of pulmonary sarcoidosis [57]. Parenchymal lung changes consistent with sarcoidosis, as determined by pulmonologists or radiologists, is one of the revised IWOS criteria for suspected OS.

Parenchymal lung changes seen in chest CT scans can be seen both in sarcoidosis and tuberculosis. In one study, parenchymal involvement among patients with uveitis was observed more frequently in patients with tuberculosis than with presumed sarcoidosis [58]. Allegri et al. reported that among adult patients with definite and presumed ocular sarcoidosis, HRCT showed purely parenchymal involvement in 40% of patients and in 13,2% parenchymal involvement was combined with hilar and/or mediastinal lymphadenomegaly [40].

#### Children > 5 years and < 5 years

The sensitivity and specificity of various findings in chest imaging modalities in diagnosis of ocular sarcoidosis in children or in Blau syndrome is unknown.

However, in children of mean age 13 years with all types of sarcoidosis, bilateral hilar lymphadenopathy was shown to be the most frequent chest X-ray manifestation (78%) [1] Parenchymal involvement with or without BHL was less frequent (16%) [1]. Also, in contrast, enhanced chest CT in children with pulmonary sarcoidosis hilar/mediastinal lymphadenopathy was the most common finding [59].

In one study, among 13 children (median age 12 years old) with sarcoidosis-associated uveitis, none of the 3 patients with definite, biopsy-proven sarcoidosis in whom chest X-ray was performed, had chest X-ray consistent with sarcoidosis [41]. In the same study, 5 patients (39%) had X-ray consistent with sarcoidosis, and were classified as presumed or probable sarcoidosis following criteria established by authors [41].

In Blau syndrome, pulmonary involvement with interstitial lung disease (ILD) is rare but can occur [31, 60]. The studies do not specify presence of BHL in Blau syndrome, although generalized lymphadenopathy was observed in 52% of patients with Blau syndrome in one study [31].

#### Conclusion chest X-ray and chest computed tomography (CT) scan

Both chest X-ray and chest CT scan play an important role in detecting BHL and parenchymal lung changes that can support diagnosis of suspected ocular sarcoidosis in adults. They were proven to be the most sensitive investigations supporting diagnosis of OS in adults. In children, who have more often extrapulmonary sarcoidosis, there is no comparative data on chest X-ray and chest CT scan contribution to diagnosis of ocular sarcoidosis.

### 18F-fluorodeoxyglucose positron emission tomography imaging (FDG PET CT) and gallium-67 scintigraphy

#### Adults

18F-fluorodeoxyglucose positron emission tomography (FDG PET CT) and Gallium-67 scintigraphy are nuclear medicine imaging methods that use radiopharmaceuticals (FDG) or radioactive isotopes to detect increased inflammatory activity. 18F-fluorodeoxyglucose positron emission tomography indicates increased glucose uptake by macrophages and lymphocytes which indicates active sites of inflammation [61].

Although FDG PET CT has gained much more attention in the research in the last decade it is still not easily accessible everywhere, where Gallium-67 scintigraphy can still have its place. FDG PET CT advantages over Gallium-67 scintigraphy are increased contrast and resolution [62]. FDG PET CT was also shown to detect more pulmonary than non-pulmonary [63] or extra thoracic [64] sarcoidosis lesions than 67 Ga citrate scintigraphy.

Neither FDG PET CT nor Gallium-67 scintigraphy is recommended in standard systemic sarcoidosis workup. However, in cardiac sarcoidosis FDG PET CT is a second choice in a lack of cardiac MRI [61, 65].

Both Gallium-67 scintigraphy and 18F-fluorodeoxyglucose positron emission tomography are included in revised criteria for ocular sarcoidosis IWOS.

**Fig. 4:**
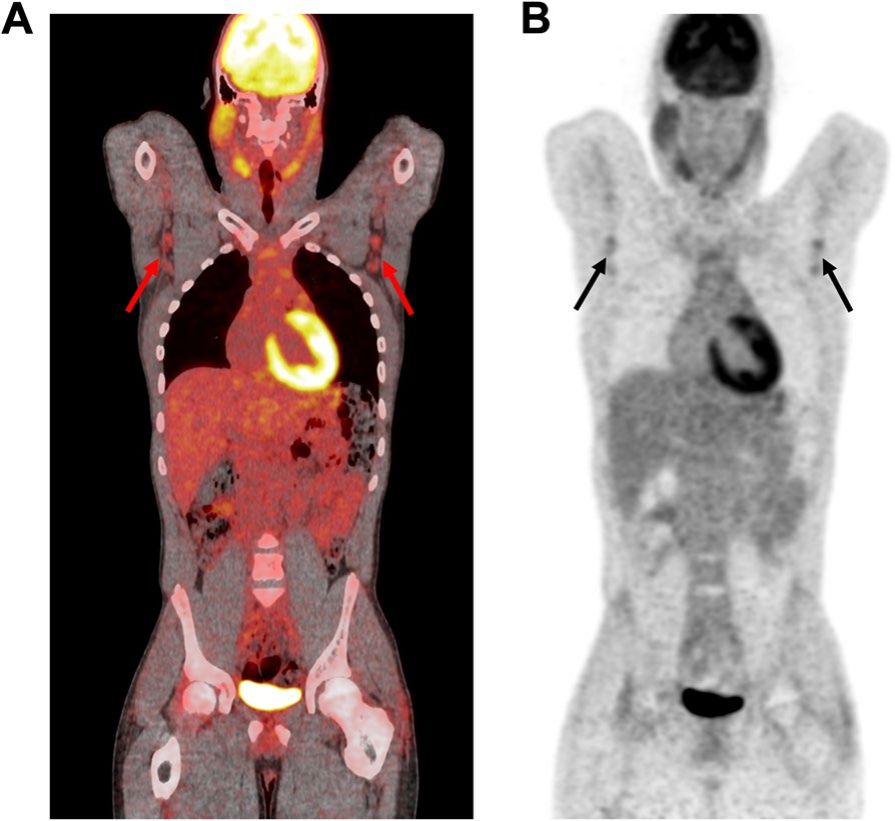
Whole body 18F-FDG PET/ ultra low dose CT in a 11 year-old patient revealed abnormal 18F-FDG uptake not specific for an active granulomatous disease. A left axillary lymph node was biopsied and definite ocular sarcoidosis was diagnosed. **A** FDG PET/CT Fusion Coronal View. **B** FDG PET coronal view. Red and black arrows: Low hypermetabolic axillary bilateral lymph nodes

FDG PET CT’s main advantage is that it is a single whole-body examination that can detect various extrapulmonary sites of sarcoidosis [66]. In FDG PET CT and in Gallium-67 scintigraphy lambda sign and panda signs are used as characteristic signs [67, 68] for diagnosis of sarcoidosis. Moreover, other sites of FDG intake can also be helpful for diagnosis by indicating locations for accessible biopsies [66, 69] (Fig. 4A, B).

Opinions vary concerning the utility of FDG PET CT in ocular sarcoidosis in adults. Chauvelot et al., showed that FDG PET CT enabled the diagnosis of intraocular sarcoidosis even in patients with a normal CT scan [70]. However, Burger et al., did not observe additional benefit of FDG PET CT over chest CT in diagnosing suspected OS [55].

FDG PET CT’s sensitivity and specificity for ocular sarcoidosis are respectively 85.7% and 95.5%. Positive and negative predictive values for FDG PET CT for ocular sarcoidosis were also calculated to be 85.7% and 95.5% [55].

As far as gallium-67 scintigraphy is concerned, there is considerably less data related to ocular sarcoidosis. The combination of elevated ACE and a positive 67GA scan increased the diagnostic specificity to 100% without affecting sensitivity (73%) in patients with suspected ocular sarcoidosis and normal chest radiographs [71].

#### Children > 5 years

FDG PET CT in pediatrics is a valuable diagnostic tool for fever of unknown origin (FUO) [72] leading to final diagnosis mostly of inflammatory (43%) or infectious (23%) origins, followed by malignancies (11%) [73]. FDG PET CT can also detect other granulomatous diseases such as intrathoracic and extra thoracic tuberculosis in children [74].

In children, a variety of strategies are possible to reduce the radiation dose while ensuring image quality [75]. These include CT attenuation correction and patient preparation.

Using ultra-low dose protocols in total body FDG PET CT is possible in children and should have special attention. Recently an ultra-low dose infection imaging using FDG PET CT was reported to be performed without sedation even in a newborn [76].

In diagnosing bilateral uveitis of undetermined origin in children, FDG PET/ ultra-low dose CT provided important information for final diagnosis in 30% patients [69]. In this study, for three pediatric patients the extra thoracic FDG intake showed biopsy accessible sites (cervical, axillary or inguinal lymph nodes) and led in 2 patients to biopsy proven, PET-CT guided ocular sarcoidosis (Fig. 4).

#### Children < 5 years

No studies were found on FDG PET CT in patients with Blau syndrome.

#### Conclusion 18F-fluorodeoxyglucose positron emission tomography imaging (FDG PET CT) and gallium-67 scintigraphy

FDG PET CT and Gallium-67 scintigraphy have their place in diagnosing OS and abnormal intake and these imaging modalities are among the revised 2019 IWOS criteria in adults. In children, who have more often extra thoracic sarcoidosis presentations, a single whole body FDG PET/ ultra-low CT could be useful for final diagnosis in indeterminate uveitis as it can indicate places accessible for biopsy that are out of scope of thoracic CT. Nowadays, many strategies can be used to reduce the radiation dose, including PET/CT with ultra-low dose CT protocol which should be of special consideration in children.

### CD4/CD8 ratio (> 3.5) in bronchoalveolar lavage fluid

#### Adults

Systemic sarcoidosis diagnosis is supported by a CD4/CD8 ratio > 3.5 and lymphocytosis > 15% in bronchoalveolar lavage (BAL) fluid. However, BAL lymphocytosis alone is not specific for sarcoidosis as it is present in many other disorders, including hypersensitivity pneumonitis, nonspecific interstitial pneumonitis, or organizing pneumonia [77, 78]. Using CD4/CD8 ratio > 3.5 increases specificity for sarcoidosis to 93–96% but still does not have high sensitivity (53 to 59%).

Other authors cited by Kraaijvanger et al. showed a wider range of BAL CD4/CD8 ratio sensitivity (54—80%) with lower specificity (59—80%) [62].

In revised IWOS criteria, elevated CD4/CD8 ratio (> 3.5) in bronchoalveolar lavage fluid (BAL) was recognised as one of systemic investigations for diagnosing suspected ocular sarcoidosis. The sensitivity and specificity of CD4/CD8 ratio (> 3.5) in BAL in diagnosing ocular sarcoidosis was not reported in the literature. However, there are studies showing its importance in diagnosing ocular sarcoidosis even in patients with normal chest imaging [79] including high-resolution computed tomography (HRCT) [80]. Another study showed that positive BAL findings were present in 67.3% of adult patients with definite and presumed ocular sarcoidosis [40].

#### Children > 5 years and < 5 years

In children as well as in adults, BAL can be performed under sedation and topical anesthesia and using flexible bronchoscopy [81]. However, serial BAL is not routinely recommended in pulmonary sarcoidosis in children in whom BAL lymphocytosis does not correlate with disease activity and treatment response [3].

There is no data on the utility of CD4/CD8 ratio (> 3.5) in BAL in ocular sarcoidosis in children or in Blau Syndrome.

#### Conclusion CD4/CD8 ratio (> 3.5) in bronchoalveolar lavage fluid

In adults, testing CD4/CD8 ratio (> 3.5) in bronchoalveolar lavage fluid (BAL) can be considered in diagnosing suspected ocular sarcoidosis, as stated in revised IWOS criteria. However, its character needing at least sedation and topic anesthesia do not place it as a first-choice diagnostic examination. Furthermore, in children, it can be even less recommended due to lack of data regarding its utility in ocular sarcoidosis.

### Serum Angiotensin Converting Enzyme (sACE)

#### Adults

ACE, studied in sarcoidosis since 1975, is the best-known serum biomarker in this disease. Serum ACE is an acid glycoprotein converting angiotensin I into angiotensin II. In the context of sarcoidosis ACE is produced by activated alveolar macrophages and correlates with granulomas burden [62]. Elevated sACE is also observed in ocular sarcoidosis, although no correlation was found between activity of sarcoidosis-associated uveitis and ACE [82].

Elevated sACE is one of the eight IWOS systemic investigations recommended as criteria for probable ocular sarcoidosis. However, it is not needed for definite or presumed ocular sarcoidosis (IWOS criteria 2019) nor for SUN Criteria for Sarcoidosis-Associated Uveitis) [7, 38]. sACE, as a sarcoidosis biomarker, is also mentioned among recent criteria of probable systemic sarcoidosis recommended by American Thoracic Society [65].

Elevated levels of sACE can be found not only in sarcoidosis but also in several other diseases, among which some can also have ocular manifestations eg. tuberculosis, leprosy, diabetes mellitus and histoplasmosis. sACE levels can be influenced by ACE inhibitors, corticosteroids use and cigarette smoking [33, 62, 83].

Notably, it was recently shown that genotype influences the sACE levels and some researchers advise to take ACE gene polymorphism into account while interpreting normal sACE levels for individuals. Taking into account the insertion (I)/deletion (D) polymorphism in the ACE gene can influence interpretation of 8,5% of measurements by either elevating or normalizing ACE values in patients with confirmed or suspected systemic sarcoidosis [84].

Sensitivity and specificity of sACE in ocular sarcoidosis varies among the studies. However, all recent studies are compatible with the fact that sACE has lower sensitivity than specificity, with sensitivity 48% and specificity 96% [50, 53, 83, 85, 86]. sACE had positive predictive value (PPV) of 44.9% and negative predictive value (NPV) 89.2% in diagnosing sarcoid uveitis [85].

To increase the sensitivity of sACE as a biomarker in ocular sarcoidosis, the combination with other biomarkers was tested leading to better sensitivity while keeping high specificity. The combinations of sACE and chest radiography, lymphopenia or sIL2R were studied and showed the following changes in sensitivity of combined examinations vs sensitivity of sACE alone. Combination of sACE and chest radiography showed increase of sensitivity (70% vs 30%) [82] as well as combination of sACE and sIL2R (75.0% vs 44.2%) [87]. Combination of sACE and lymphopenia showed an increase of sensitivity to 18.9% when compared with sensitivity of lymphopenia alone (15.3%) but showed a decrease in sensitivity when compared with sensitivity of sACE alone (45.8%) [85].

The standard cut off value of sACE for adults is 68 U/L. However, the optimal cutoff point for sACE levels in the population with uveitis was calculated to be 51 U/L [82]. The normal standard values may vary among the regions eg. in Japan standard sACE normal range is 7.0–25.0 IU/L [88] with recent proposition to change the cut-off value to 17.7 IU/L which would increase sensitivity of detecting sarcoidosis to 67.0% in Japan [88].

In a recent study of ocular sarcoidosis patients, the mean serum levels of ACE were 49.17 ± 29 IU/L versus 27.4 ± 15.34 IU/L in the control group of non-granulomatous (i.e., non-sarcoidosis) uveitis patients [50].

#### Children > 5 years and < 5 years

Although, there is no study that determines sensitivity or specificity of sACE in children with ocular sarcoidosis, this biomarker is used in pediatric clinical practice. From a study in Louisiana on childhood sarcoidosis (*n* = 27) we know that ACE was elevated in 74% of sarcoidosis pediatric patients and among all patients in the study 77% children had uveitis [20]. Another study showed that among 13 children with probable, presumed, or definite sarcoidosis, 6 patients had elevated ACE levels [41].

In the 1980s, Baarsma and co found that ACE were age dependent [89]. In accordance with this finding, nowadays pediatric sACE normal values are (29–112 U/I) and they differ from those used for adults (20–70 U/I) [50].

In one study, all (*n* = 9) pediatric patients diagnosed with sarcoidosis-associated uveitis had elevated ACE levels [42].

#### Conclusion serum Angiotensin Converting Enzyme (sACE)

sACE is a biomarker used in ocular and systemic sarcoidosis in adults and children. It is among the revised IWOS criteria for diagnosing suspected sarcoidosis. Its sensitivity in ocular sarcoidosis is not very high but it can be increased if combined with other diagnostic tests. The recent studies show that ACE gene polymorphism can affect the interpretation of sACE normal values. In children ACE normal values are higher than for adults. There are few studies reporting ACE in pediatric systemic or ocular sarcoidosis.

### Serum lysozyme

#### Adults

Lysozyme is a bacteriolytic enzyme, a part of innate immunity. It degrades peptidoglycans that are mostly present in the walls of gram positive bacteria. In sarcoidosis, lysozyme is produced by monocyte macrophage systems and epithelioid cells, and is involved in granuloma formation [62].

In ocular sarcoidosis, elevated serum lysozyme has been among the diagnostic criteria indicated by IWOS since 2006 and in the reviewed 2019 IWOS criteria it is even stated as a criterion separate from ACE. However, in the systemic sarcoidosis criteria of American Thoracic Society from 2020 [65] lysozyme is not mentioned among the criteria.

The frequency of elevated lysozyme in sarcoidosis patients varies among the studies, 18,8% [90]—79,1% [91] and its level correlates with the number of organs involved [91]. In ocular sarcoidosis (biopsy-proven or BHL positive or suspected) lysozyme levels were shown to be elevated in 59,4% of patients [50]. Both lysozyme and ACE were elevated in 24,3% patients and ACE alone was elevated in only 5,4% of patients [50]. Another study showed that 61% of patients with ocular sarcoidosis (biopsy-proven or BHL positive) had elevated sACE or lysozyme or both [39].

Moreover, similarly to systemic sarcoidosis [91] in ocular sarcoidosis there is a correlation between serum lysozyme levels and disease burden [92]. In ocular sarcoidosis, lower lysozyme levels were observed in patients with biopsied sub-centimetric mediastinal lymph nodes in comparison to patients that had bigger (≥ 1 cm) lymph nodes [93].

Rarely lysozyme can be elevated in ocular infections but it was not elevated in autoimmune ocular disorders other than presumed ocular sarcoidosis. Serum lysozyme was found to be rarely elevated in presumed latent ocular tuberculosis or presumed latent syphilis [94]. However, it was not elevated in patients with ocular involvement of other autoimmune diseases such Behçet’s disease and ankylosing spondylitis [94].

In ocular sarcoidosis, lysozyme has a sensitivity of 83.7% and a specificity of 90% [50], which contrasts with sACE’s low specificity. The authors of this recent study concluded that lysozyme was found to be more useful than ACE as a laboratory test to support the diagnosis of ocular sarcoidosis [50].

Normal lysozyme values are 9.6–17.1 mg/L for all ages and mean serum lysozyme levels was 39.92 ± 55.5 mg/L in the ocular sarcoidosis group versus 10.5 ± 5.8 mg/L (*p* ≤ 0.0013) in the control group (*n* = 30) [50].

#### Children > 5 years and < 5 years

There are no studies stating lysozyme sensitivity and specificity in ocular sarcoidosis in children. In one study, among 13 children with probable, presumed, or definite sarcoidosis 5 patients had elevated lysozyme levels [41].

Serum lysozyme normal values in children are the same as for adults (9.6–17.1 mg/L) [50], although initially reported in the 80 s to be age dependent in ocular sarcoidosis [89].

#### Conclusion serum lysozyme

Serum lysozyme is among systemic investigations criteria for diagnosing probable OS according to revised IWOS. It has high sensitivity in diagnosing OS and some studies find it even more useful than sACE. It was shown to be elevated in some pediatric patients with OS.

### Lymphopenia

Lymphopenia or lymphocytopenia is the blood lymphocyte count below an age-appropriate reference.

Lymphopenia can occur in many conditions and among them are: steroid therapy, autoimmune disorders like lupus erythematosus, infectious diseases like tuberculosis, AIDS and viral hepatitis. Significant lymphopenia (below 1000 cells/μL) was shown to be an independent predictor of sarcoidosis in new patients presenting with uveitis [95].

In IWOS 2019 criteria lymphopenia (< 1000 cells/μL) was added among the diagnostic criteria of ocular sarcoidosis, as the peripheral blood lymphocyte count is a simple, non-invasive test that is readily performed in patients with uveitis (IWOS 2019).

Lymphopenia was observed in 35.1% patients with ocular sarcoidosis in a German study [86] as well as in 26.8% patients with sarcoidosis-associated uveitis in the UK [95].

Although, different laboratories may have slightly different normal values, in many publications the normal lymphocyte count in adults is 1000 to 4800/mcL (1 to 4.8 × 10^9^/L) and in children younger than 2 years 3000 to 9500/mcL (3 to 9.5 × 10^9^/L) [96]. For children aged 6 years, lymphopenia is recognized if lymphocyte count is less than 1500/mcL (1.5 × 10^9^/L). The range of normal lymphocyte values in teenagers (12-18y) approach adults’ norms (1,1—4.5 × 10^9^/L) [97] but should be verified with local laboratory values. Notably, some authors distinguish severe lymphopenia < 1000/mcL (< 1,0 × 10^9^/L) and relative lymphopenia < 1500/mcL (< 1,5 × 10^9^/L) [98].

Sensitivity of lymphopenia as a biomarker in diagnosing uveitis can vary and can depend on the choice of lymphocyte cut-off value. There are two recent studies that calculated sensitivity and specificity of lymphopenia in diagnosing sarcoid uveitis but the results differ. When considering the cut-off value of severe lymphopenia (< 1,0 × 10^9^/L), the lymphopenia sensitivity in diagnosing sarcoid uveitis was low (15.3%) with high specificity (96.7%) [85]. However, in another study a cut-off value close to relative lymphopenia (< 1.47 × 10^9^/L) gave higher sensitivity (75%) with lower specificity (77%) [82]. In the latter study, authors justify the choice of lymphocyte cut-off < 1.47 × 10^9^/L with the highest Youden index, a marker of the performance of a diagnostic test, for diagnosing sarcoidosis-associated uveitis.

#### In children > 5 years and < 5 years

There is no study showing the frequency of lymphopenia in children with ocular sarcoidosis or with patients with Blau syndrome.

#### Conclusion lymphopenia

Lymphopenia is of importance in diagnosing suspected ocular sarcoidosis and can be detected by a simple and routinely done blood test. It is one of the criteria in revised IWOS for probable OS in adults. There is no data concerning the role of lymphopenia in diagnosing OS in children or in Blau syndrome.

### Serum Soluble Interleukin 2 Receptor (sIL2R)

sIL2R is a circulating form of membrane receptor for IL2 which is shed from the surface of activated Th1 cells. Activated Th1 cells are involved in formation and perpetuation of granuloma [62] including those sarcoidosis-associated. Elevated sIL2R is not specific for sarcoidosis and can also be present in other granulomatous diseases, hematological malignancies, and various autoimmune disorders [62].

sIL2R has been studied as a potential biomarker in systemic sarcoidosis. However, its role is not yet well established and is not mentioned in the diagnostic guidelines for lung sarcoidosis [65]. sIL2R was neither included in the reviewed IWOS ocular sarcoidosis criteria as it was not used widely enough at the time [7]. However, there is accumulating data supporting the diagnostic value of sIL2R in ocular sarcoidosis.

sIL2R was found to be elevated in 69,2% [87]- 76.2% [86] patients with ocular sarcoidosis. sIL2R can be elevated also in 5.4% patients without sarcoid uveitis and in 16.7% of patients with primary intraocular lymphoma (PIOL) [87]. Another study found that serum sIL-2R levels can be elevated in patients with HLA-B27–associated and varicella-zoster virus–associated uveitis, however, with serum sIL2R levels lower than in sarcoidosis-associated uveitis [82].

Recently, several studies evaluating sensitivity and/or specificity of sIL2R in ocular sarcoidosis diagnosis have been published. Two of them showed high specificity of sIL2R in ocular sarcoidosis in the Japanese population [53, 87]. sIL2R sensitivity in ocular sarcoidosis diagnosis ranged from 69.2% [87] to 76.4% [53] and specificity from 93.0% [87] to 93.8% [53]. The third study showed sIL2R sensitivity of 70.6% in definite and presumed ocular sarcoidosis in the German population [86]. Interestingly, an earlier German study, considering not only definite, presumed, but also probable and possible OS, showed sIL2R sensitivity of 98% and specificity of 94% [99]. All these data suggest that sIL2R shows higher sensitivity and specificity in ocular sarcoidosis diagnosis than sACE.

Measurements of Youden index were performed in some recent studies in ocular sarcoidosis. Youden index of sIL2R (0.70) in Japanese population was higher than for other biomarkers in ocular sarcoidosis eg. ACE (0.35), KL-6 (0.26), and calcemia (0.07). The authors suggested that it can indicate superior utility of sIL2R among serum biomarkers in diagnosing ocular sarcoidosis [53]. Although the highest Youden index for sIL2R (0.45) found by the Dutch group was lower than by the Japanese group, the researchers conclusion was similar underlying the usefulness of sIL2R for diagnosing sarcoidosis in patients with uveitis [82]. Another earlier European German study, including not only definite, presumed but also probable and possible OS, calculated the Youden index of sIL2R to be 0,92 [99].

The optimal sIL2R cutoff value in detecting ocular sarcoidosis found by Dutch researchers was 4000 pg/mL [82] and a Japanese group used sIL2R cutoff values > 543 U/mL [87]. Mean serum sIL-2R levels were 834.5 ± 486.7 U/mL in patients with sarcoid uveitis, which was higher than in patients with non-sarcoid uveitis 313.0 ± 127.7 U/mL [87]. Another group measured average serum sIL-2R levels in the presumed disease group to be 1325.2 U/mL [86].

#### Children > 5 years and < 5 years

There are no studies on sIL2 in sarcoidosis nor in ocular sarcoidosis in the pediatric population.

#### Conclusion Serum Soluble Interleukin 2 Receptor (sIL2R)

In conclusion, serum sIL2R is a promising biomarker in ocular sarcoidosis with many recent studies carried out in adult patients. Several independent groups showed that the sensitivity of serum sIL2R in diagnosing ocular sarcoidosis is higher than the sensitivity of sACE. However, there are no related studies in the pediatric population.

### Other potential biomarkers tested in ocular sarcoidosis

#### Krebs von den Lungen-6 (KL-6)

Krebs von den Lungen-6 (KL-6), which is a mucin-like glycoprotein produced by pneumocytes or bronchiolar epithelial cells, was proposed as a marker of pulmonary cells injury or inflammation. Its elevated serum levels were found in sarcoidosis but also in idiopathic pulmonary fibrosis and other interstitial lung diseases [62]. KL-6 was tested in the Japanese population as a biomarker for ocular sarcoidosis in adults. It showed sensitivity of 26.3% with good specificity (96.2%)[53]. In the same study it showed lower sensitivity than sIL2R (76,4%) and ACE (37,7%) but higher than Ca (11,8%).

KL-6 was not tested in children with sarcoidosis nor with ocular sarcoidosis. There are several studies testing KL-6 in pediatric pulmonary diseases. One study showed KL-6 as a useful biomarker for pediatric patients with connective tissue disease accompanied by interstitial lung disease [100].

#### Hypercalcemia

Hypercalcemia occurs in up to 4% of the population in many health conditions, including sarcoidosis, tuberculosis and lymphomas. In systemic sarcoidosis it is present in 7–18% patients [77, 101], although in Japan it was observed even in 35% patients [90]. Hypercalcemia in sarcoidosis is a result of ectopic production of calcitriol 1,25(OH)2D3 by activated macrophages within granulomas [77].

For diagnosing ocular sarcoidosis in adults in Japan, elevated calcium (Ca) levels had rather low sensitivity (11.8%) with good specificity (95.1%)[53].

There are no studies concerning hypercalcemia in children with ocular sarcoidosis. One case report found hypercalcemia useful in diagnosing uncommon onset sarcoidosis in a 14-year-old child without ocular involvement [102].

#### Polyclonal antibody

Polyclonal antibody activity testing bases on an observation that there is a compensatory increase of immunoglobulins as a result of decrease of T cell activity in sarcoidosis. The serologies of four herpesviruses (EBV, CMV, HSV, VZV) were used to calculate the polyclonal activation ratio [50].

One study showed that polyclonal antibody testing has high sensitivity (70%) and specificity (90.4%) in ocular sarcoidosis [50].

There is no data concerning children with ocular sarcoidosis and polyclonal antibody activity.

#### CXCL9 and CXCL10

Several chemokines produced by monocyte-macrophage cell lineage were shown to be elevated in sarcoidosis and to play many different roles including T-cell attraction and promotion of Th1/Th17 differentiation [62].

In diagnosed or suspected ocular sarcoidosis, serum levels of both CXCL9 and CXCL10 were markedly elevated and correlated with ocular disease activity and ACE level [103]. Chemokines were also tested in aqueous humor in patients with uveitis and it was found that CXCL13 were significantly higher in granulomatous uveitis, including sarcoidosis [104].

There are no further studies concerning sensitivity or specificity of chemokines in ocular sarcoidosis neither in children nor in adults.

#### Liver enzymes

Liver enzymes are not included in the revised IWOS criteria for diagnosing ocular sarcoidosis, in contrast to previous criteria from 2009. In sarcoidosis liver involvement was shown to be present in 2.5–11.5% patients [66] or even up to 35% of patients [77]. However, in ocular sarcoidosis elevated hepatic enzymes were rarely present, only in 5% of patients [39].

#### B-cell activating factor (BAFF)

B-cell activating factor (BAFF) is a cytokine of the TNF family that plays a vital role in the growth and function of B cells [62]. Elevated BAFF levels are not specific for sarcoidosis and have also been found in other immunomodulatory diseases like systemic lupus erythematosus (SLE) and rheumatoid arthritis (RA) [62].

One study has shown that in sarcoidosis patients elevated serum BAFF show no significant difference between patients with anterior uveitis and those without ocular involvement [105].

There are no further studies concerning sensitivity or specificity of serum BAFF in ocular sarcoidosis neither in children nor in adults.

#### Serum microRNA (miRNA)

MiRNAs are small noncoding RNAs that regulate gene expression at the post-transcriptional level and are among the circulating cell-free nucleic acids released into the serum/plasma by various tissues and cells [106]. MiRNAs were proposed as biomarkers for the diagnosis of non-infectious uveitis [107].

Very recently miRNA microarrays (GeneChip®) were used to investigate serum miRNA profiles in patients with ocular sarcoidosis and patients with intraocular inflammation that appears typical for patients diagnosed with ocular sarcoidosis, but in whom the results of laboratory testing do not fulfill the IWOS diagnostic criteria (suspected ocular sarcoidosis). The study demonstrated a high overlap of the differential expression of serum miRNAs in these two groups of patients [106].

Although it is only one, small and preliminary study that does not indicate sensitivity and specificity for ocular sarcoidosis diagnosis, it can open a way to establish diagnoses in idiopathic uveitis patients. There are no studies on serum miRNA in children with ocular sarcoidosis or BS.

#### Conclusion other potential biomarkers tested in ocular sarcoidosis

Among other biomarkers tested in ocular sarcoidosis such as KL-6, hypercalcemia, polyclonal antibody activity, chemokines,serum BAFF, and serum microRNA. The latter.

seems most promising, although further studies are needed. There is no data related to these biomarkers in pediatric patients with ocular sarcoidosis.

### Chitotriosidase and other biomarkers tested in systemic sarcoidosis but not yet in ocular sarcoidosis

Biomarkers tested in pulmonary sarcoidosis and not yet in ocular sarcoidosis are: chitotriosidase, serum neuron-specific enolase (NSE), Serum Amyloid A (SAA), neopterin, YKL40, sCD16 and CCL18 [62, 108, 109]. Among them chitotriosidase is very promising and in some specialties now often used as a biomarker.

Chitotriosidase is a chitinase involved in defense against chitin-containing pathogens [110]. The enzyme has been found elevated in serum and bronchoalveolar lavage (BAL) of patients with sarcoidosis in comparison to patients with other interstitial lung diseases, pulmonary tuberculosis and healthy controls [111, 112]. In sarcoidosis patients, chitotriosidase showed higher sensitivity and specificity than other biomarkers, including angiotensin converting enzyme (ACE), lysozyme and soluble IL-2 receptor. It has been found increased in active sarcoidosis patients [108, 113–116].

### Ocular biopsies

#### Adults

Ocular biopsies can be divided into intraocular biopsies including vitreous fluid and ocular adnexa biopsies such as conjunctival biopsy.

Intraocular biopsies are really performed because of its invasive character. However, it is worth noticing that the CD4/CD8 ratio in the vitreous fluid showed high sensitivity (100%) and specificity (96.3%) for the diagnosis of ocular sarcoidosis in one study [117]. Another multicenter, prospective study confirmed that vitreous CD4/CD8 or CD4 + measurements are higher in ocular sarcoidosis than in other uveitis etiologies [118].

In very rare cases, choroidal or subretinal biopsies in 27-Gauge pars plana vitrectomies are performed [119]. This is limited to atypical, progressing and sight-threatening lesions where other diagnostic methods were inconclusive [120]. A new alternative to histological biopsy of the uvea for diagnosing ocular sarcoidosis is histological detection of epithelioid granuloma and epithelioid cells in liquid-based cytology from vitreous body specimens and in the cell block procedure from vitreous cell components in an intraocular irrigating solution [121].

Conjunctival biopsy is less invasive, however, not commonly used. Conjunctival biopsy may be positive in approximately 50% of patients with sarcoidosis [122]. The direct data on its sensitivity and specificity in patients with uveitis were not found. However, there is some data concerning conjunctival biopsies in patients with uveitis. One study on a group of 10 patients with ocular findings like those of multifocal choroiditis with panuveitis showed that non-directed conjunctival biopsy disclosed non-caseating granulomata in seven of them [123]. In another study, in patients with uveitis suspected to be secondary to sarcoidosis, directed biopsy of conjunctival follicles was found to be positive for sarcoidosis in up to 63% of patients [124].

Furthermore, several studies mention conditions for increased positive conjunctival biopsy yield in diagnosis of sarcoidosis. Spaide et al. reported that the conjunctival biopsy was more likely to be positive in patients with conjunctival follicles, ocular abnormalities consistent with sarcoidosis, and in patients with pulmonary infiltrates on chest X-ray [125]. To increase the positive yield of conjunctival biopsies it was also recommended to perform bilateral conjunctival biopsies from multiple levels of the tissue [122] or to use a multi-plane technique instead of standard sectioning technique [124].

Conjunctival biopsy is very interesting from a cost-effectiveness point of view. While positive results of conjunctival biopsy were like mediastinoscopy, the cost of conjunctival biopsy was ten times lower.

#### Children > 5 and < 5 years

The data on conjunctival biopsies in children in sarcoidosis is limited to case reports. One case report presented a 10-year-old female patient with conjunctival deposits but with no other ocular or systemic complaints, in whom conjunctival biopsies proved systemic sarcoidosis [126]. Another case report showed noncaseating lipogranulomatous subconjunctival nodules as a novel presenting finding in Blau syndrome in the absence of uveitis [127].

In Blau syndrome, conjunctival biopsies proved conjunctival granulomas in 2 adults and one 10-year-old child [44].

#### Conclusion ocular biopsies

Although highly sensitive and specific for sarcoidosis, the CD4/CD8 ratio in the vitreous fluid, can rarely be performed in everyday clinics. Conjunctival biopsy is a minimal invasive and cost-effective examen that should not be forgotten especially in patients with suspected ocular sarcoidosis and conjunctival follicles.

### Genetic testing

In case of the typical triad of Blau Syndrome (arthritis, rash, uveitis) genetic testing can be performed not only in children below 5 years of age but also in older patients. Although the median age at onset of eye involvement in Blau Syndrome is 5 years (range 0,5–48 years) [26] in studies the final diagnosis of Blau Syndrome was made even in the adults, sometimes proving a long diagnostic delay.

To diagnose Blau syndrome or EOS, confirmation of mutation in NOD2 gene is needed and genetic counseling for patients is recommended. Traditionally, Blau syndrome is recognised if there is a family history of the disease and EOS if the mutation is sporadic [21]. However, authors of some recent publications [26–29] use the name of Blau Syndrome for both familial and sporadic mutations.

Mutations that are most frequently present in Blau Syndrome are R334W and R334Q [21, 26] and several more mutations have been identified in recent years [26, 29, 51, 128, 129]. Mutations in Blau Syndrome are autosomal dominant, gain of function mutations.

Diagnosing ocular sarcoidosis with onset in adulthood does not need genetic testing and is based on international criteria (Tables 1 and 2) [7, 38].

The diagnostic criteria for ocular sarcoidosis in children are not yet clearly stated.

Of interest, single nucleotide polymorphisms (SNPs) in HLA and non-HLA genes are known to be associated with ocular sarcoidosis. HLA-DRB1*04:01 is known to be associated with OS in European-Americans [130]. Variants of several non-HLA genes were also shown to be associated with increased risk of sarcoidosis-associated uveitis or OS: RAB23, ANXA11 [131] and MAGI1 [130], CHF [132], HSP-70/Hom [133], IL23R gene [134].

## Conclusions

In this article, we review the clinical manifestations, differential diagnosis and diagnostic modalities of sarcoidosis-associated uveitis/ocular sarcoidosis in adults and children. We also refer to Blau Syndrome and Early Onset Sarcoidosis, in which uveitis typically starts in children under 5 years of age.

Clinical features of sarcoidosis-associated uveitis (SUN criteria) or ocular sarcoidosis (revised IWOS criteria) are well studied in adults but data concerning children older than 5 years is sparse.

In adults and children over 5 years of age ocular sarcoidosis is typically bilateral with panuveitis or anterior uveitis as a predominant site of inflammation depending on the study. Panuveitis is more frequent in Asian populations and most common in BS/EOS. In adults, granulomatous uveitis was observed in about half of the patients, often with mutton-fat keratic precipitates. In children over 5 years old it is possible that non-granulomatous uveitis is more frequent. In all age groups, multifocal choroiditis is the most frequent posterior presentation, followed by periphlebitis. In BS/EOS posterior synechiae and peripapillary nodules are more common than in other forms of sarcoidosis.

Differential diagnosis should exclude especially ocular tuberculosis and syphilis. In children ocular sarcoidosis should be differentiated with JIA-associated uveitis and TINU.

There are many diagnostic modalities supporting the diagnosis of ocular sarcoidosis but none of them is ideal or self-sufficient. Definite ocular sarcoidosis diagnosis can be stated only in case of biopsy proven lesions with compatible ocular involvement. For presumed and probable ocular sarcoidosis diagnosis performing systemic imaging or checking for serum biomarkers is needed.

If biopsy is not possible, bilateral hilar lymphadenopathy (BHL) is the next most important test. CT has higher sensitivity than chest X-ray in detecting BHL in adults. Serum ACE and lysozyme are well documented as ocular sarcoidosis biomarkers. Availability of lymphopenia is its major advantage although its sensitivity is not high. There are several very promising studies on sIL2R as an ocular sarcoidosis biomarker showing its higher sensitivity even than ACE. In some patients, conjunctival biopsies or vitreous biopsies can be considered.

There is very little data concerning the utility of diagnostic tests in ocular sarcoidosis in children, therefore a detailed comparison with adults is not possible. In contrast to adults, in children, extrapulmonary sarcoidosis is more frequent. Therefore, searching for biopsy accessible peripheral lymph nodes by the means of FDG PET/CT (with ultra-low CT protocol) could be an interesting option especially in children with bilateral uveitis with negative chest imaging. Blau Syndrome and EOS are, respectively, the familial and sporadic forms of the same monogenic autoinflammatory disease that needs genetic testing to be confirmed. A search for NOD2 gene mutation should be considered if a typical triad of arthritis, rash, uveitis is present.

## Data Availability

All data generated or analysed during this study are included in this published article.

## Abbreviations

IWOS: International Workshop on Ocular Sarcoidosis
SUN: Standardization of Uveitis Nomenclature
BS: Blau syndrome
EOS: Early-onset sarcoidosis
CT: Computed tomography
FDG PET-CT: 18F-fluorodeoxyglucose positron emission tomography - computed tomography
NOD2: Nucleotide-binding oligomerization domain-containing protein 2
ACE: Angiotensin converting enzyme
IL2R: Soluble interleukin 2 receptor
CARD15: Caspase recruitment domain-containing protein 15
APMPPE: Acute posterior multifocal placoid pigment epitheliopathy
VKH: Vogt-Koyanagi-Harada
TINU: Tubulointerstitial nephritis and uveitis
JIA: Juvenile idiopathic arthritis
JRA: Juvenile rheumatoid arthritis
BHL: Bilateral hilar lymphadenopathy
HLA-B27: Human leukocyte antigen
OS: Ocular sarcoidosis
ILD: Interstitial lung disease
MRI: Magnetic resonance imaging
CD4/CD8: Clusters of differentiation 4/ clusters of differentiation 8
BAL: Bronchoalveolar lavage
HRCT: High-resolution computed tomography
sACE: Serum angiotensin converting enzyme
PPV: Positive predictive value
NPV: Negative predictive value
PIOL: Primary intraocular lymphoma
KL-6: Krebs von den Lungen-6
CXCL9: C-X-C motif chemokine ligand 9
BAFF: B-cell activating factor
TNF: Tumor necrosis factor
miRNA: MicroRNA

## References

[1] Hoffmann AL, Milman N, Byg KE (2004). Childhood sarcoidosis in Denmark 1979–1994: incidence, clinical features and laboratory results at presentation in 48 children. Acta Paediatr.

[2] Melani AS, Bigliazzi C, Cimmino FA, Bergantini L, Bargagli E (2021). A comprehensive review of sarcoidosis treatment for pulmonologists. Pulm Ther.

[3] Shetty AK, Gedalia A (2008). Childhood sarcoidosis: a rare but fascinating disorder. Pediatr Rheumatol Online J.

[4] Pasadhika S, Rosenbaum JT (2015). Ocular sarcoidosis. Clin Chest Med.

[5] Heiligenhaus A, Wefelmeyer D, Wefelmeyer E, Rosel M, Schrenk M (2011). The eye as a common site for the early clinical manifestation of sarcoidosis. Ophthalmic Res.

[6] Rothova A, Alberts C, Glasius E, Kijlstra A, Buitenhuis HJ, Breebaart AC (1989). Risk factors for ocular sarcoidosis. Doc Ophthalmol.

[7] Mochizuki M, Smith JR, Takase H, Kaburaki T, Acharya NR, Rao NA, International Workshop on Ocular Sarcoidosis Study G (2019). Revised criteria of International Workshop on Ocular Sarcoidosis (IWOS) for the diagnosis of ocular sarcoidosis. Br J Ophthalmol.

[8] Millward K, Fiddler CA, Thillai M (2021). Update on sarcoidosis guidelines. Curr Opin Pulm Med.

[9] Judson MA, Costabel U, Drent M, Wells A, Maier L, Koth L, Shigemitsu H, Culver DA, Gelfand J, Valeyre D, Sweiss N, Crouser E, Morgenthau AS, Lower EE, Azuma A, Ishihara M, Morimoto S, Tetsuo Yamaguchi T, Shijubo N, Grutters JC, Rosenbach M, Li HP, Rottoli P, Inoue Y, Prasse A, Baughman RP, Baughman RP, Organ Assessment Instrument Investigators TW (2014). The WASOG Sarcoidosis Organ Assessment Instrument: an update of a previous clinical tool. Sarcoidosis Vasc Diffuse Lung Dis.

[10] Fraga RC, Kakizaki P, Valente NYS, Portocarrero LKL, Teixeira MFS, Senise PF (2017). Do you know this syndrome? Heerfordt-Waldenstrom syndrome. An Bras Dermatol.

[11] Teirstein AS, Judson MA, Baughman RP, Rossman MD, Yeager H, Moller DR, Case Control Etiologic Study of Sarcoidosis Writing G (2005). The spectrum of biopsy sites for the diagnosis of sarcoidosis. Sarcoidosis Vasc Diffuse Lung Dis.

[12] Shah KK, Pritt BS, Alexander MP (2017). Histopathologic review of granulomatous inflammation. J Clin Tuberc Other Mycobact Dis.

[13] Prasse A (2016). The diagnosis, differential diagnosis, and treatment of sarcoidosis. Dtsch Arztebl Int.

[14] Bernardinello N, Petrarulo S, Balestro E, Cocconcelli E, Veltkamp M, Spagnolo P (2021) Pulmonary sarcoidosis: diagnosis and differential diagnosis. Diagnostics (Basel) 11(9). 10.3390/diagnostics11091558 10.3390/diagnostics11091558 PMC8472810 34573900

[15] Lee GM, Pope K, Meek L, Chung JH, Hobbs SB, Walker CM (2020). Sarcoidosis: a diagnosis of exclusion. AJR Am J Roentgenol.

[16] Strickland-Marmol LB, Fessler RG, Rojiani AM (2000). Necrotizing sarcoid granulomatosis mimicking an intracranial neoplasm: clinicopathologic features and review of the literature. Mod Pathol.

[17] Ganeshan D, Menias CO, Lubner MG, Pickhardt PJ, Sandrasegaran K, Bhalla S (2018). Sarcoidosis from head to toe: what the radiologist needs to know. Radiographics.

[18] Chiu B, Chan J, Das S, Alshamma Z, Sergi C (2019) Pediatric Sarcoidosis: A Review with Emphasis on Early Onset and High-Risk Sarcoidosis and Diagnostic Challenges. Diagnostics (Basel) 9(4). 10.3390/diagnostics9040160 10.3390/diagnostics9040160 PMC6963233 31731423

[19] Spagnolo P, Rossi G, Trisolini R, Sverzellati N, Baughman RP, Wells AU (2018). Pulmonary sarcoidosis. Lancet Respir Med.

[20] Gedalia A, Khan TA, Shetty AK, Dimitriades VR, Espinoza LR (2016). Childhood sarcoidosis: Louisiana experience. Clin Rheumatol.

[21] Wouters CH, Maes A, Foley KP, Bertin J, Rose CD (2014). Blau syndrome, the prototypic auto-inflammatory granulomatous disease. Pediatr Rheumatol Online J.

[22] Milman N, Hoffmann AL (2008). Childhood sarcoidosis: long-term follow-up. Eur Respir J.

[23] Ellis JC, Faber BG, Uri IF, Emerson SJ (2020). Early onset sarcoidosis (Blau syndrome): erosive and often misdiagnosed. Rheumatology (Oxford).

[24] Caso F, Costa L, Rigante D, Vitale A, Cimaz R, Lucherini OM, Sfriso P, Verrecchia E, Tognon S, Bascherini V, Galeazzi M, Punzi L, Cantarini L (2014). Caveats and truths in genetic, clinical, autoimmune and autoinflammatory issues in Blau syndrome and early onset sarcoidosis. Autoimmun Rev.

[25] Kanazawa N, Okafuji I, Kambe N, Nishikomori R, Nakata-Hizume M, Nagai S, Fuji A, Yuasa T, Manki A, Sakurai Y, Nakajima M, Kobayashi H, Fujiwara I, Tsutsumi H, Utani A, Nishigori C, Heike T, Nakahata T, Miyachi Y (2005). Early-onset sarcoidosis and CARD15 mutations with constitutive nuclear factor-kappaB activation: common genetic etiology with Blau syndrome. Blood.

[26] Sarens IL, Casteels I, Anton J, Bader-Meunier B, Brissaud P, Chedeville G, Cimaz R, Dick AD, Espada G, Fernandez-Martin J, Guly CM, Hachulla E, Harjacek M, Khubchandani R, Mackensen F, Merino R, Modesto C, Naranjo A, Oliveira-Knupp S, Ozen S, Pajot C, Ramanan AV, Russo R, Susic G, Thatayatikom A, Thomee C, Vastert S, Bertin J, Arostegui JI, Rose CD, Wouters CH (2018). Blau syndrome-associated uveitis: preliminary results from an international prospective interventional case series. Am J Ophthalmol.

[27] Kumrah R, Pilania RK, Menia NK, Rawat A, Sharma J, Gupta A, Vignesh P, Jindal AK, Rikhi R, Agarwal A, Gupta V, Singh S, Suri D (2022). Blau syndrome: lessons learned in a tertiary care centre at Chandigarh. North India. Front Immunol.

[28] Agarwal A, Karande S (2022). Blau syndrome: an under-reported condition in India?. J Postgrad Med.

[29] Matsuda T, Kambe N, Ueki Y, Kanazawa N, Izawa K, Honda Y, Kawakami A, Takei S, Tonomura K, Inoue M, Kobayashi H, Okafuji I, Sakurai Y, Kato N, Maruyama Y, Inoue Y, Otsubo Y, Makino T, Okada S, Kobayashi I, Yashiro M, Ito S, Fujii H, Kondo Y, Okamoto N, Ito S, Iwata N, Kaneko U, Doi M, Hosokawa J, Ohara O, Saito MK, Nishikomori R, JSIAD Pmit, JSIAD Pmit (2020). Clinical characteristics and treatment of 50 cases of Blau syndrome in Japan confirmed by genetic analysis of the NOD2 mutation. Ann Rheum Dis.

[30] Yao Q, Zhou L, Cusumano P, Bose N, Piliang M, Jayakar B, Su LC, Shen B (2011). A new category of autoinflammatory disease associated with NOD2 gene mutations. Arthritis Res Ther.

[31] Rose CD, Pans S, Casteels I, Anton J, Bader-Meunier B, Brissaud P, Cimaz R, Espada G, Fernandez-Martin J, Hachulla E, Harjacek M, Khubchandani R, Mackensen F, Merino R, Naranjo A, Oliveira-Knupp S, Pajot C, Russo R, Thomee C, Vastert S, Wulffraat N, Arostegui JI, Foley KP, Bertin J, Wouters CH (2015). Blau syndrome: cross-sectional data from a multicentre study of clinical, radiological and functional outcomes. Rheumatology (Oxford).

[32] Sahin N, Cicek SO, Kisaarslan AP, Gunduz Z, Poyrazoglu MH, Dusunsel R (2021). Unexpected condition in a rare disease: encephalopathy in early-onset sarcoidosis. Turk J Pediatr.

[33] Rothova A (2000). Ocular involvement in sarcoidosis. Br J Ophthalmol.

[34] Coulon C, Kodjikian L, Rochepeau C, Perard L, Jardel S, Burillon C, Broussolle C, Jamilloux Y, Seve P (2019). Ethnicity and association with ocular, systemic manifestations and prognosis in 194 patients with sarcoid uveitis. Graefes Arch Clin Exp Ophthalmol.

[35] Niederer RL, Ma SP, Wilsher ML, Ali NQ, Sims JL, Tomkins-Netzer O, Lightman SL, Lim LL (2021). Systemic associations of sarcoid uveitis: correlation with uveitis phenotype and ethnicity. Am J Ophthalmol.

[36] Han YS, Rivera-Grana E, Salek S, Rosenbaum JT (2018). Distinguishing uveitis secondary to sarcoidosis from idiopathic disease: cardiac implications. JAMA Ophthalmol.

[37] Jamilloux Y, Kodjikian L, Broussolle C, Seve P (2014). Sarcoidosis and uveitis. Autoimmun Rev.

[38] Standardization of Uveitis Nomenclature Working G,  (2021). Classification criteria for sarcoidosis-associated uveitis. Am J Ophthalmol.

[39] Acharya NR, Browne EN, Rao N, Mochizuki M, International Ocular Sarcoidosis Working G (2018). Distinguishing features of ocular sarcoidosis in an international cohort of uveitis patients. Ophthalmology.

[40] Allegri P, Olivari S, Rissotto F, Rissotto R (2022) Sarcoid uveitis: an intriguing challenger. Medicina (Kaunas) 58(7). 10.3390/medicina58070898 10.3390/medicina58070898 PMC9316395 35888617

[41] Choi DE, Birnbaum AD, Oh F, Tessler HH, Goldstein DA (2011). Pediatric uveitis secondary to probable, presumed, and biopsy-proven sarcoidosis. J Pediatr Ophthalmol Strabismus.

[42] Morelle G, Gueudry J, Uettwiller F, Wouters C, Bader-Meunier B, Robert MP, Monnet D, Bodaghi B, Grall-Lerosey M, Quartier P (2019). Chronic and recurrent non-infectious paediatric-onset uveitis: a French cohort. RMD Open.

[43] Waduthantri SMS, Chee Sp F (2021). Pediatric uveitis and scleritis in a multi-ethnic asian population. Ocul Immunol Inflamm.

[44] Babu K, Rao AP (2021). Clinical profile in genetically proven blau syndrome: a case series from South India. Ocul Immunol Inflamm.

[45] Scifo L, Willermain F, Postelmans L, Pozdzik A, Lolin Sekelj K, Zampieri M, de Jong C, Makhoul D (2022). Subclinical choroidal inflammation revealed by indocyanine green angiography in tubulointerstitial nephritis and uveitis syndrome. Ocul Immunol Inflamm.

[46] Hien DL, Onghanseng N, Ngoc TTT, Hwang JJ, Pham BH, Doan HL, Nguyen HV, Halim MS, Uludag G, Sepah YJ, Do DV, Nguyen QD (2020). Yet another case of ocular sarcoidosis. Am J Ophthalmol Case Rep.

[47] Karpathiou G, Batistatou A, Boglou P, Stefanou D, Froudarakis ME (2018). Necrotizing sarcoid granulomatosis: a distinctive form of pulmonary granulomatous disease. Clin Respir J.

[48] Testi I, Tognon MS, Gupta V (2019). Diagnostic challenges in granulomatous uveitis: tuberculosis or sarcoidosis?. Ocul Immunol Inflamm.

[49] Keenan JD, Tessler HH, Goldstein DA (2008). Granulomatous inflammation in juvenile idiopathic arthritis-associated uveitis. J AAPOS.

[50] Papasavvas I, Herbort CP (2021). Granulomatous features in juvenile idiopathic arthritis-associated uveitis is not a rare occurrence. Clin Ophthalmol.

[51] Okazaki F, Wakiguchi H, Korenaga Y, Nakamura T, Yasudo H, Uchi S, Yanai R, Asano N, Hoshii Y, Tanabe T, Izawa K, Honda Y, Nishikomori R, Uchida K, Eishi Y, Ohga S, Hasegawa S (2021). A novel mutation in early-onset sarcoidosis/Blau syndrome: an association with Propionibacterium acnes. Pediatr Rheumatol Online J.

[52] Babu K, Shukla SB, Philips M (2017). High resolution chest computerized tomography in the diagnosis of ocular sarcoidosis in a high TB endemic population. Ocul Immunol Inflamm.

[53] Suzuki K, Namba K, Mizuuchi K, Iwata D, Ito T, Hase K, Kitaichi N, Ishida S (2021). Validation of systemic parameters for the diagnosis of ocular sarcoidosis. Jpn J Ophthalmol.

[54] Grumet P, Kodjikian L, de Parisot A, Errera MH, Sedira N, Heron E, Perard L, Cornut PL, Schneider C, Riviere S, Olle P, Pugnet G, Cathebras P, Manoli P, Bodaghi B, Saadoun D, Baillif S, Tieulie N, Andre M, Chiambaretta F, Bonin N, Bielefeld P, Bron A, Mouriaux F, Bienvenu B, Vicente S, Bin S, Labetoulle M, Broussolle C, Jamilloux Y, Decullier E, Seve P, group U (2018). Contribution of diagnostic tests for the etiological assessment of uveitis, data from the ULISSE study (Uveitis: Clinical and medicoeconomic evaluation of a standardized strategy of the etiological diagnosis). Autoimmun Rev.

[55] Burger C, Holness JL, Smit DP, Griffith-Richards S, Koegelenberg CFN, Ellmann A (2021). The role of (18) F-FDG PET/CT in suspected intraocular sarcoidosis and tuberculosis. Ocul Immunol Inflamm.

[56] Takayama K, Harimoto K, Sato T, Sakurai Y, Taguchi M, Kanda T, Takeuchi M (2018). Age-related differences in the clinical features of ocular sarcoidosis. PLoS One.

[57] Criado E, Sanchez M, Ramirez J, Arguis P, de Caralt TM, Perea RJ, Xaubet A (2010). Pulmonary sarcoidosis: typical and atypical manifestations at high-resolution CT with pathologic correlation. Radiographics.

[58] Bansal R, Gupta A, Agarwal R, Dogra M, Bhutani G, Gupta V, Dogra MR, Katoch D, Aggarwal AN, Sharma A, Nijhawan R, Nada R, Nahar Saikia U, Dey P, Behera D (2019). Role of CT chest and cytology in differentiating tuberculosis from presumed sarcoidosis in uveitis. Ocul Immunol Inflamm.

[59] Gorkem SB, Kose S, Lee EY, Doganay S, Coskun AS, Kose M (2017). Thoracic MRI evaluation of sarcoidosis in children. Pediatr Pulmonol.

[60] Kaufman KP, Becker ML (2021). Distinguishing blau syndrome from systemic sarcoidosis. Curr Allergy Asthma Rep.

[61] Casali M, Lauri C, Altini C, Bertagna F, Cassarino G, Cistaro A, Erba AP, Ferrari C, Mainolfi CG, Palucci A, Prandini N, Baldari S, Bartoli F, Bartolomei M, D'Antonio A, Dondi F, Gandolfo P, Giordano A, Laudicella R, Massollo M, Nieri A, Piccardo A, Vendramin L, Muratore F, Lavelli V, Albano D, Burroni L, Cuocolo A, Evangelista L, Lazzeri E, Quartuccio N, Rossi B, Rubini G, Sollini M, Versari A, Signore A (2021). State of the art of (18)F-FDG PET/CT application in inflammation and infection: a guide for image acquisition and interpretation. Clin Transl Imaging.

[62] Kraaijvanger R, Janssen Bonas M, Vorselaars ADM, Veltkamp M (2020). Biomarkers in the diagnosis and prognosis of sarcoidosis: current use and future prospects. Front Immunol.

[63] Prager E, Wehrschuetz M, Bisail B, Woltsche M, Schwarz T, Lanz H, Sorantin E, Aigner RM (2008). Comparison of 18F-FDG and 67Ga-citrate in sarcoidosis imaging. Nuklearmedizin.

[64] Nishiyama Y, Yamamoto Y, Fukunaga K, Takinami H, Iwado Y, Satoh K, Ohkawa M (2006). Comparative evaluation of 18F-FDG PET and 67Ga scintigraphy in patients with sarcoidosis. J Nucl Med.

[65] Crouser ED, Maier LA, Wilson KC, Bonham CA, Morgenthau AS, Patterson KC, Abston E, Bernstein RC, Blankstein R, Chen ES, Culver DA, Drake W, Drent M, Gerke AK, Ghobrial M, Govender P, Hamzeh N, James WE, Judson MA, Kellermeyer L, Knight S, Koth LL, Poletti V, Raman SV, Tukey MH, Westney GE, Baughman RP (2020). Diagnosis and detection of sarcoidosis. An official american thoracic society clinical practice guideline. Am J Respir Crit Care Med.

[66] Valeyre D, Jeny F, Rotenberg C, Bouvry D, Uzunhan Y, Seve P, Nunes H, Bernaudin JF (2021). How to tackle the diagnosis and treatment in the diverse scenarios of extrapulmonary sarcoidosis. Adv Ther.

[67] Sulavik SB, Spencer RP, Palestro CJ, Swyer AJ, Teirstein AS, Goldsmith SJ (1993). Specificity and sensitivity of distinctive chest radiographic and/or 67Ga images in the noninvasive diagnosis of sarcoidosis. Chest.

[68] Shim H, Joo J, Choi HJ, Hyun H, Gerbaudo VH, Kim CK (2016). Lack of increased FDG uptake in the lacrimal and salivary glands in patients with sarcoidosis and potential underlying mechanism. Clin Nucl Med.

[69] Bazewicz M, Makhoul D, Goffin L, El Mouden J, Judice MRL, Caspers L, Draganova D, Postelmans L, Garcia C, Willermain F (2023). Clinical utility of (18)F-FDG PET/CT in the work-up of children with uveitis. Ocul Immunol Inflamm.

[70] Chauvelot P, Skanjeti A, Jamilloux Y, de Parisot A, Broussolle C, Denis P, Ramackers JM, Giammarile F, Kodjikian L, Seve P (2019). (18)F-fluorodeoxyglucose positron emission tomography is useful for the diagnosis of intraocular sarcoidosis in patients with a normal CT scan. Br J Ophthalmol.

[71] Power WJ, Neves RA, Rodriguez A, Pedroza-Seres M, Foster CS (1995). The value of combined serum angiotensin-converting enzyme and gallium scan in diagnosing ocular sarcoidosis. Ophthalmology.

[72] Pijl JP, Nienhuis PH, Kwee TC, Glaudemans A, Slart R, Gormsen LC (2021). Limitations and pitfalls of FDG-PET/CT in infection and inflammation. Semin Nucl Med.

[73] Nygaard U, Larsen LV, Vissing NH, von Linstow ML, Myrup C, Berthelsen AK, Poulsen A, Borgwardt L (2022). Unexplained fever in children-Benefits and challenges of FDG-PET/CT. Acta Paediatr.

[74] Pelletier-Galarneau M, Martineau P, Zuckier LS, Pham X, Lambert R, Turpin S (2017). (18)F-FDG-PET/CT Imaging of thoracic and extrathoracic tuberculosis in children. Semin Nucl Med.

[75] Parisi MT, Bermo MS, Alessio AM, Sharp SE, Gelfand MJ, Shulkin BL (2017). Optimization of pediatric PET/CT. Semin Nucl Med.

[76] van Rijsewijk ND, van Leer B, Ivashchenko OV, Scholvinck EH, van den Heuvel F, van Snick JH, Slart R, Noordzij W, Glaudemans A (2023). Ultra-low dose infection imaging of a newborn without sedation using long axial field-of-view PET/CT. Eur J Nucl Med Mol Imaging.

[77] Seve P, Pacheco Y, Durupt F, Jamilloux Y, Gerfaud-Valentin M, Isaac S, Boussel L, Calender A, Androdias G, Valeyre D, El Jammal T (2021) Sarcoidosis: a clinical overview from symptoms to diagnosis. Cells 10(4). 10.3390/cells10040766 10.3390/cells10040766 PMC8066110 33807303

[78] Costabel U, Bonella F, Ohshimo S, Guzman J (2010). Diagnostic modalities in sarcoidosis: BAL, EBUS, and PET. Semin Respir Crit Care Med.

[79] Caspers L, Makhoul D, Ebraert H, Michel O, Willermain F (2014). Clinical manifestations of patients with intraocular inflammation and positive QuantiFERON-TB gold in-tube test in a country nonendemic for tuberculosis. Am J Ophthalmol.

[80] Takahashi T, Azuma A, Abe S, Kawanami O, Ohara K, Kudoh S (2001). Significance of lymphocytosis in bronchoalveolar lavage in suspected ocular sarcoidosis. Eur Respir J.

[81] de Blic J, Midulla F, Barbato A, Clement A, Dab I, Eber E, Green C, Grigg J, Kotecha S, Kurland G, Pohunek P, Ratjen F, Rossi G (2000). Bronchoalveolar lavage in children. ERS task force on bronchoalveolar lavage in children European Respiratory Society. Eur Respir J.

[82] Groen-Hakan F, Eurelings L, ten Berge JC, van Laar J, Ramakers CRB, Dik WA, Rothova A (2017). Diagnostic value of serum-soluble interleukin 2 receptor levels vs angiotensin-converting enzyme in patients with sarcoidosis-associated uveitis. JAMA Ophthalmol.

[83] Hu YQ, Lv CY, Cui A (2022). Pulmonary sarcoidosis: a novel sequelae of drug reaction with eosinophilia and systemic symptoms: a case report. World J Clin Cases.

[84] Kruit A, Grutters JC, Ruven HJ, van Moorsel CH, Weiskirchen R, Mengsteab S, van den Bosch JM (2006). Transforming growth factor-beta gene polymorphisms in sarcoidosis patients with and without fibrosis. Chest.

[85] Cotte P, Pradat P, Kodjikian L, Jamilloux Y, Seve P (2021). Diagnostic value of lymphopaenia and elevated serum ACE in patients with uveitis. Br J Ophthalmol.

[86] Zur Bonsen LS, Pohlmann D, Rubsam A, Pleyer U (2021) Findings and graduation of sarcoidosis-related uveitis: a single-center study. Cells 11(1). 10.3390/cells11010089 10.3390/cells11010089 PMC8750073 35011651

[87] Ishihara M, Meguro A, Ishido M, Takeuchi M, Shibuya E, Mizuki N (2020). Usefulness of combined measurement of serum soluble IL-2R and angiotensin-converting enzyme in the detection of uveitis associated with japanese sarcoidosis. Clin Ophthalmol.

[88] Baba Y, Kubo T, Yamanaka S, Ochi Y, Hirota T, Yamasaki N, Ohnishi H, Kubota T, Yokoyama A, Kitaoka H (2019). Reconsideration of the cut-off value of angiotensin-converting enzyme for screening of sarcoidosis in Japanese patients. J Cardiol.

[89] Baarsma GS, La Hey E, Glasius E, de Vries J, Kijlstra A (1987). The predictive value of serum angiotensin converting enzyme and lysozyme levels in the diagnosis of ocular sarcoidosis. Am J Ophthalmol.

[90] Yamanouchi Y, Sawahata M, Sakamoto N, Hisata S, Shijubo N, Konno S, Yamaguchi T, Watanabe M, Kawashima H, Suzuki T, Bando M, Hagiwara K (2020). Characteristics of 68 patients with clinically proven sarcoidosis based on the Japan Society of Sarcoidosis and Other Granulomatous Disorders 2015 criteria. Respir Investig.

[91] Tomita H, Sato S, Matsuda R, Sugiura Y, Kawaguchi H, Niimi T, Yoshida S, Morishita M (1999). Serum lysozyme levels and clinical features of sarcoidosis. Lung.

[92] Weinberg RS, Tessler HH (1976). Serum lysozyme in sarcoid uveitis. Am J Ophthalmol.

[93] Bolletta E, Macchioni P, Citriniti G, Mastrofilippo V, Aldigeri R, De Simone L, Gozzi F, Adani C, Sangiovanni A, Posarelli C, Figus M, Muratore F, Pipitone N, Salvarani C, Cimino L (2022). Clinical features and prevalence of spondyloarthritis in a cohort of Italian patients presenting with acute nongranulomatous anterior uveitis. J Immunol Res.

[94] Sahin O, Ziaei A (2016). Clinical and laboratory characteristics of ocular syphilis, co-infection, and therapy response. Clin Ophthalmol.

[95] Jones NP, Tsierkezou L, Patton N (2016). Lymphopenia as a predictor of sarcoidosis in patients with uveitis. Br J Ophthalmol.

[96] https://www.nbt.nhs.uk/sites/default/files/Childrens%20FBC%20Reference%20Ranges.pdf

[97] David A (2019) Immunology

[98] Zidar DA, Al-Kindi SG, Liu Y, Krieger NI, Perzynski AT, Osnard M, Nmai C, Anthony DD, Lederman MM, Freeman ML, Bonomo RA, Simon DI, Dalton JE (2019). Association of lymphopenia with risk of mortality among adults in the US general population. JAMA Netw Open.

[99] Gundlach E, Hoffmann MM, Prasse A, Heinzelmann S, Ness T (2016). Interleukin-2 receptor and angiotensin-converting enzyme as markers for ocular sarcoidosis. PLoS One.

[100] Kilinc AA, Arslan A, Yildiz M, Kucur M, Adrovic A, Barut K, Sahin S, Cokugras H, Kasapcopur O (2020). Serum KL-6 level as a biomarker of interstitial lung disease in childhood connective tissue diseases: a pilot study. Rheumatol Int.

[101] Chebbi D, Marzouk S, Snoussi M, Regaieg N, Gouiaa N, Ben Salah R, Damak C, Frikha F, Boudawara T, Bahloul Z (2021). Pure extra-thoracic sarcoidosis: about 24 cases. Rom J Intern Med.

[102] Marginean CO, Melit LE, Grigorescu G, Puiac C, Simu I (2020). Hypercalcemia, an important puzzle piece in uncommon onset pediatric sarcoidosis-a case report and a review of the literature. Front Pediatr.

[103] Takeuchi T, Tsutsumi O, Ikezuki Y, Kamei Y, Osuga Y, Fujiwara T, Takai Y, Momoeda M, Yano T, Taketani Y (2006). Elevated serum bisphenol A levels under hyperandrogenic conditions may be caused by decreased UDP-glucuronosyltransferase activity. Endocr J.

[104] El-Asrar AMA, Berghmans N, Al-Obeidan SA, Gikandi PW, Opdenakker G, Van Damme J, Struyf S (2018). Differential CXC and CX3C chemokine expression profiles in aqueous humor of patients with specific endogenous uveitic entities. Invest Ophthalmol Vis Sci.

[105] Hashemzadeh K, Fatemipour M, Zahra Mirfeizi S, Jokar M, Shariati Sarabi Z, Hatef Fard MR, Rafatpanah H, Khodashahi M (2021). Serum B cell activating factor (BAFF) and sarcoidosis activity. Arch Rheumatol.

[106] Saito S, Keino H, Takasaki I, Abe S, Kohno H, Ichihara K, Hayashi I, Nakayama M, Tsuboshita Y, Miyoshi S, Okamoto S, Okada AA (2022) Comparative Analysis of serum microRNA in diagnosed ocular sarcoidosis versus idiopathic uveitis with ocular manifestations of sarcoidosis. Int J Mol Sci 23 (18). 10.3390/ijms231810749 10.3390/ijms231810749 PMC9506523 36142662

[107] Asakage M, Usui Y, Nezu N, Shimizu H, Tsubota K, Yamakawa N, Takanashi M, Kuroda M, Goto H (2020). Comprehensive miRNA analysis using serum from patients with noninfectious uveitis. Invest Ophthalmol Vis Sci.

[108] Ramos-Casals M, Retamozo S, Siso-Almirall A, Perez-Alvarez R, Pallares L, Brito-Zeron P (2019). Clinically-useful serum biomarkers for diagnosis and prognosis of sarcoidosis. Expert Rev Clin Immunol.

[109] Sunaga N, Koga Y, Hachisu Y, Yamaguchi K, Aikawa M, Kasahara N, Miura Y, Tsurumaki H, Yatomi M, Sakurai R, Maeno T, Hisada T (2022). Role of neuron-specific enolase in the diagnosis and disease monitoring of sarcoidosis. Can Respir J.

[110] Funkhouser JD, Aronson NN (2007). Chitinase family GH18: evolutionary insights from the genomic history of a diverse protein family. BMC Evol Biol.

[111] Bargagli E, Bennett D, Maggiorelli C, Di Sipio P, Margollicci M, Bianchi N, Rottoli P (2013). Human chitotriosidase: a sensitive biomarker of sarcoidosis. J Clin Immunol.

[112] Bargagli E, Rottoli P (2007). Serum chitotriosidase activity in sarcoidosis patients. Rheumatol Int.

[113] Bennett D, Cameli P, Lanzarone N, Carobene L, Bianchi N, Fui A, Rizzi L, Bergantini L, Cillis G, d'Alessandro M, Mazzei MA, Refini RM, Sestini P, Bargagli E, Rottoli P (2020). Chitotriosidase: a biomarker of activity and severity in patients with sarcoidosis. Respir Res.

[114] Lopes MC, Amadeu TP, Ribeiro-Alves M, da Costa CH, Rodrigues LS, Bessa EJC, Bruno LP, Lopes AJ, Rufino R (2019). Identification of active sarcoidosis using chitotriosidase and angiotensin-converting enzyme. Lung.

[115] Malkova A, Zinchenko Y, Starshinova A, Kudlay D, Kudryavtsev I, Glushkova A, Yablonskiy P, Shoenfeld Y (2022). Sarcoidosis: progression to the chronic stage and pathogenic based treatment (narrative review). Front Med (Lausanne).

[116] Di Francesco AM, Verrecchia E, Sicignano LL, Massaro MG, Antuzzi D, Covino M, Pasciuto G, Richeldi L, Manna R (2021) The use of chitotriosidase as a marker of active sarcoidosis and in the diagnosis of Fever of Unknown Origin (FUO). J Clin Med 10(22). 10.3390/jcm10225283 10.3390/jcm10225283 PMC8619698 34830565

[117] Kojima K, Maruyama K, Inaba T, Nagata K, Yasuhara T, Yoneda K, Sugita S, Mochizuki M, Kinoshita S (2012). The CD4/CD8 ratio in vitreous fluid is of high diagnostic value in sarcoidosis. Ophthalmology.

[118] Maruyama K, Inaba T, Sugita S, Ichinohasama R, Nagata K, Kinoshita S, Mochizuki M, Nakazawa T (2017). Comprehensive analysis of vitreous specimens for uveitis classification: a prospective multicentre observational study. BMJ Open.

[119] Grewal DS, Cummings TJ, Mruthyunjaya P (2017). Outcomes of 27-gauge vitrectomy-assisted choroidal and subretinal biopsy. Ophthalmic Surg Lasers Imaging Retina.

[120] Daley JR, Cherepanoff S, Heydon PG, Fung AT (2022). Subretinal peripapillary biopsy-proven sarcoidosis: a case report. Int J Retina Vitreous.

[121] Ohe R, Kaneko Y, Namba H, Nishi K, Goto JI, Futakuchi M, Nishitsuka K (2022). Utility of liquid-based cytology and cell block procedure obtained by vitrectomy to diagnose ocular sarcoidosis-the significance of epithelioid granuloma and epithelioid cells. Clin Ophthalmol.

[122] Leavitt JA, Campbell RJ (1998). Cost-effectiveness in the diagnosis of sarcoidosis: the conjunctival biopsy. Eye (Lond).

[123] Hershey JM, Pulido JS, Folberg R, Folk JC, Massicotte SJ (1994). Non-caseating conjunctival granulomas in patients with multifocal choroiditis and panuveitis. Ophthalmology.

[124] Bui KM, Garcia-Gonzalez JM, Patel SS, Lin AY, Edward DP, Goldstein DA (2014). Directed conjunctival biopsy and impact of histologic sectioning methodology on the diagnosis of ocular sarcoidosis. J Ophthalmic Inflamm Infect.

[125] Spaide RF, Ward DL (1990). Conjunctival biopsy in the diagnosis of sarcoidosis. Br J Ophthalmol.

[126] Caca I, Unlu K, Buyukbayram H, Ari S (2006). Conjunctival deposits as the first sign of systemic sarcoidosis in a pediatric patient. Eur J Ophthalmol.

[127] Ahmad M, Hermanson ME, Enzenauer R, Palestine A, Lin C, Meeks N, McCourt E (2017). Lipogranulomatous subconjunctival nodules: a novel presentation in Blau syndrome. J AAPOS.

[128] Kim W, Park E, Ahn YH, Lee JM, Kang HG, Kim BJ, Ha IS, Cheong HI (2016). A familial case of Blau syndrome caused by a novel NOD2 genetic mutation. Korean J Pediatr.

[129] Rodrigues FG, Petrushkin H, Webster AR, Bickerstaff M, Moraitis E, Rowczenio D, Arostegui JI, Westcott M (2021). A novel pathogenic NOD2 variant in a mother and daughter with blau syndrome. Ophthalmic Genet.

[130] Garman L, Pezant N, Pastori A, Savoy KA, Li C, Levin AM, Iannuzzi MC, Rybicki BA, Adrianto I, Montgomery CG (2021). Genome-wide association study of ocular sarcoidosis confirms HLA associations and implicates barrier function and autoimmunity in African Americans. Ocul Immunol Inflamm.

[131] Davoudi S, Chang VS, Navarro-Gomez D, Stanwyck LK, Sevgi DD, Papavasileiou E, Ren A, Uchiyama E, Sullivan L, Lobo AM, Papaliodis GN, Sobrin L (2018). Association of genetic variants in RAB23 and ANXA11 with uveitis in sarcoidosis. Mol Vis.

[132] Thompson IA, Liu B, Sen HN, Jiao X, Katamay R, Li Z, Hu M, Hejtmancik F, Nussenblatt RB (2013). Association of complement factor H tyrosine 402 histidine genotype with posterior involvement in sarcoid-related uveitis. Am J Ophthalmol.

[133] Spagnolo P, du Bois RM (2007). Genetics of sarcoidosis. Clin Dermatol.

[134] Kim HS, Choi D, Lim LL, Allada G, Smith JR, Austin CR, Doyle TM, Goodwin KA, Rosenbaum JT, Martin TM (2011). Association of interleukin 23 receptor gene with sarcoidosis. Dis Markers.

